# DIXDC1 Promotes Lymphatic Metastasis and Resistance to Cuproptosis in Bladder Cancer Through Mediating DLAT

**DOI:** 10.1002/advs.77435

**Published:** 2026-08-27

**Authors:** Hongqiong Li, Liang Cheng, Xiaole Lu, Haonan Dong, Shi Fu, Hongjin Shi, Mingsheng Liu, Chenwei Yang, Yuhang Huang, Zehua Chen, Yawei Zhang, Kaiwen Jie, Xiaoyue Zhang, Xiang Kui, Xinni Ye, Siting Chen, Xu Chen, Haifeng Wang

**Affiliations:** ^1^ Department of Urology The Second Affiliated Hospital of Kunming Medical University Kunming Yunnan People's Republic of China; ^2^ Department of Urology Sun Yat‐sen Memorial Hospital Sun Yat‐sen University Guangzhou Guangdong People's Republic of China; ^3^ Guangdong Provincial Key Laboratory of Malignant Tumor Epigenetics and Gene Regulation Sun Yat‐sen Memorial Hospital State Key Laboratory of Oncology in South China Guangzhou Guangdong People's Republic of China; ^4^ Guangdong Provincial Clinical Research Center for Urological Diseases Guangzhou Guangdong People's Republic of China; ^5^ Department of Urology Qujing Affiliated Hospital of Kunming Medical University Qujing Yunnan People's Republic of China; ^6^ Department of Pathology The Second Affiliated Hospital of Kunming Medical University Kunming Yunnan People's Republic of China

**Keywords:** bladder cancer, cuproptosis, DIXDC1, DLAT, lymphatic metastasis

## Abstract

Lymph node metastasis (LN) represents a major clinical challenge in bladder cancer (BCa) and is associated with dismal prognosis; however, the underlying molecular drivers remain incompletely understood. Here, we demonstrate that DIX domain‐containing protein 1 (DIXDC1) is significantly upregulated in LN‐metastatic BCa tissues and is associated with unfavorable clinical outcomes. Functionally, DIXDC1 enhances BCa cell migration, invasion, proliferation, and lymph node dissemination in vivo. Mechanistically, DIXDC1 directly binds to dihydrolipoamide S‐acetyltransferase (DLAT), a key enzyme in the tricarboxylic acid cycle and a central effector of cuproptosis. This interaction impedes ubiquitin‐proteasome‐mediated degradation of DLAT, thereby stabilizing DLAT protein levels. Through a DLAT‐dependent pathway, DIXDC1 further augments the mRNA stability of the oncogenes *NEK7* and *CCNE2*, fueling tumor progression. Importantly, DIXDC1 shields BCa cells from copper‐induced DLAT oligomerization and confers marked resistance to Elesclomol‐Cu (ES‐Cu)‐triggered cuproptosis. Depletion of DIXDC1 profoundly sensitizes tumors to cuproptosis in vivo. Collectively, our study unveils the DIXDC1‐DLAT axis as a pivotal regulator that coordinately drives metastatic progression and suppresses copper‐induced cell death, offering a promising therapeutic target for advanced BCa.

## Graphical abstract

DIXDC1 drives bladder cancer progression by binding and stabilizing DLAT. This axis enhances NEK7 and CCNE2 mRNA stability to promote proliferation and lymphatic metastasis, while limiting copper‐induced DLAT oligomerization to reduce cuproptosis sensitivity, identifying DIXDC1 as a potential target linking metastasis with cuproptosis resistance.

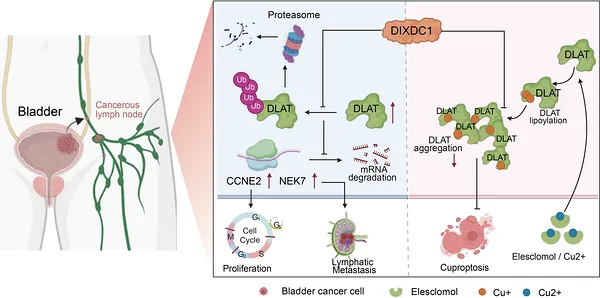

## 1 Introduction

Bladder cancer (BCa) represents a significant global health issue, with an estimated 613 000 new cases and 220 000 fatalities occurring annually [1, 2]. Lymphatic metastasis is the most frequent and earliest form of metastasis in BCa. Data from 2025 indicate that once BCa progresses to lymph node (LN) metastasis, the five‐year survival rate drops dramatically from 70% to 30%, with the mortality risk increasing by 2.25 times [3, 4, 5]. Therefore, elucidating the molecular mechanisms of lymphatic metastasis is essential for developing effective treatments.

Metabolic plasticity is a pivotal driver of cancer progression, conferring the adaptive capacity necessary to navigate the metastatic cascade [6, 7]. To satisfy the heightened bioenergetic requirements of metastatic dissemination, BCa cells often undergo a metabolic shift toward enhanced mitochondrial oxidative metabolism and increased reliance on the tricarboxylic acid (TCA) cycle [8, 9, 10]. Nevertheless, this metabolic shift exposes an intrinsic vulnerability to cuproptosis—a form of copper‐induced death triggered by the toxic oligomerization of the TCA gatekeeper, dihydrolipoamide S‐acetyltransferase (DLAT) [11, 12]. This creates a fundamental metabolic‐survival paradox, where the very enzyme essential for fueling metastasis also serves as a lethal death‐sensor [13, 14]. How metastatic cells reconcile these conflicting programs during BCa progression remains a critical unanswered question.

DIX domain‐containing protein 1 (DIXDC1) is a multifunctional scaffolding protein that has been classically characterized as a key organizer of the Wnt/β‐catenin signaling cascade during tumor development and progression [15, 16, 17, 18]. However, the functional repertoire of DIXDC1 beyond classical signal transduction remains largely unexplored. While the chaperone network provides canonical defense against protein aggregation [19, 20], whether DIXDC1 leverages its unique polymerizing architecture to physically shield metabolic enzymes from the toxic aggregation that triggers cuproptosis has not been established. Identifying whether DIXDC1 synchronizes bioenergetic requirements with cuproptosis resistance and invasive signaling represents a fundamental challenge in understanding BCa lymphatic metastasis.

In this study, we identified DIXDC1 as a key regulator that integrates invasive signaling and metabolic survival during BCa lymphatic metastasis. Mechanistically, we demonstrated that DIXDC1 bound to and stabilized the DLAT protein by preventing its degradation. Importantly, this stabilization enabled DLAT to enhance the mRNA stability of *NEK7* and *CCNE2*, thereby promoting lymphatic metastasis. Furthermore, we reveal that DIXDC1 physically blocks the toxic aggregation of DLAT, protecting cancer cells from cuproptosis. Our findings establish a previously unrecognized DIXDC1‐DLAT axis that resolves the conflict between metabolic demand and cell death, offering a new strategy for treating metastatic BCa.

## 2 Results

### 2.1 DIXDC1 is Overexpressed and Associated With Poor Prognosis in BCa

DIXDC1 has been reported to facilitate tumor cell proliferation and metastatic progression in multiple malignancies [15, 21, 22]; however, its specific role and underlying mechanisms in BCa remain to be fully clarified. To investigate the expression profile and clinical relevance of DIXDC1 in BCa, we assessed its protein expression in two independent clinical cohorts via IHC. DIXDC1 protein expression was significantly increased in BCa tissues compared with paired adjacent normal tissues (NAT) across both cohorts, and was further elevated in lymph node‐positive tumors, muscle‐invasive BCa (MIBC), and high‐grade tumors relative to their corresponding counterparts (Figure 1A–C,E,F; Figure S1A,B). Consistently, transcriptomic analyses of TCGA, GSE13507, and GSE87304 datasets revealed that DIXDC1 mRNA expression was elevated in lymph node‐positive vs. lymph node‐negative samples, MIBC vs. non‐muscle‐invasive BCa (NMIBC) samples, high‐grade vs. low‐grade samples, and advanced T3‐T4 vs. T1‐T2 stage tumors, indicating a positive correlation with tumor aggressiveness (Figure 1D,G; Figure S1C–E). In addition, DIXDC1 protein levels were significantly higher in BCa tissues from patients with distant metastasis (M1) than in those from patients without metastasis (M0) in both clinical cohorts (Figure S1F,G). Kaplan‐Meier survival analyses further revealed that high DIXDC1 expression was associated with significantly shorter overall survival and disease‐free survival in both the clinical cohorts and the TCGA cohort (Figure 1H–K and Figure S1H,I). Taken together, elevated DIXDC1 expression is associated with aggressive tumor progression, increased lymphatic and distant metastasis, and unfavorable clinical outcomes in bladder cancer patients, supporting its potential as a prognostic biomarker.

**FIGURE 1:**
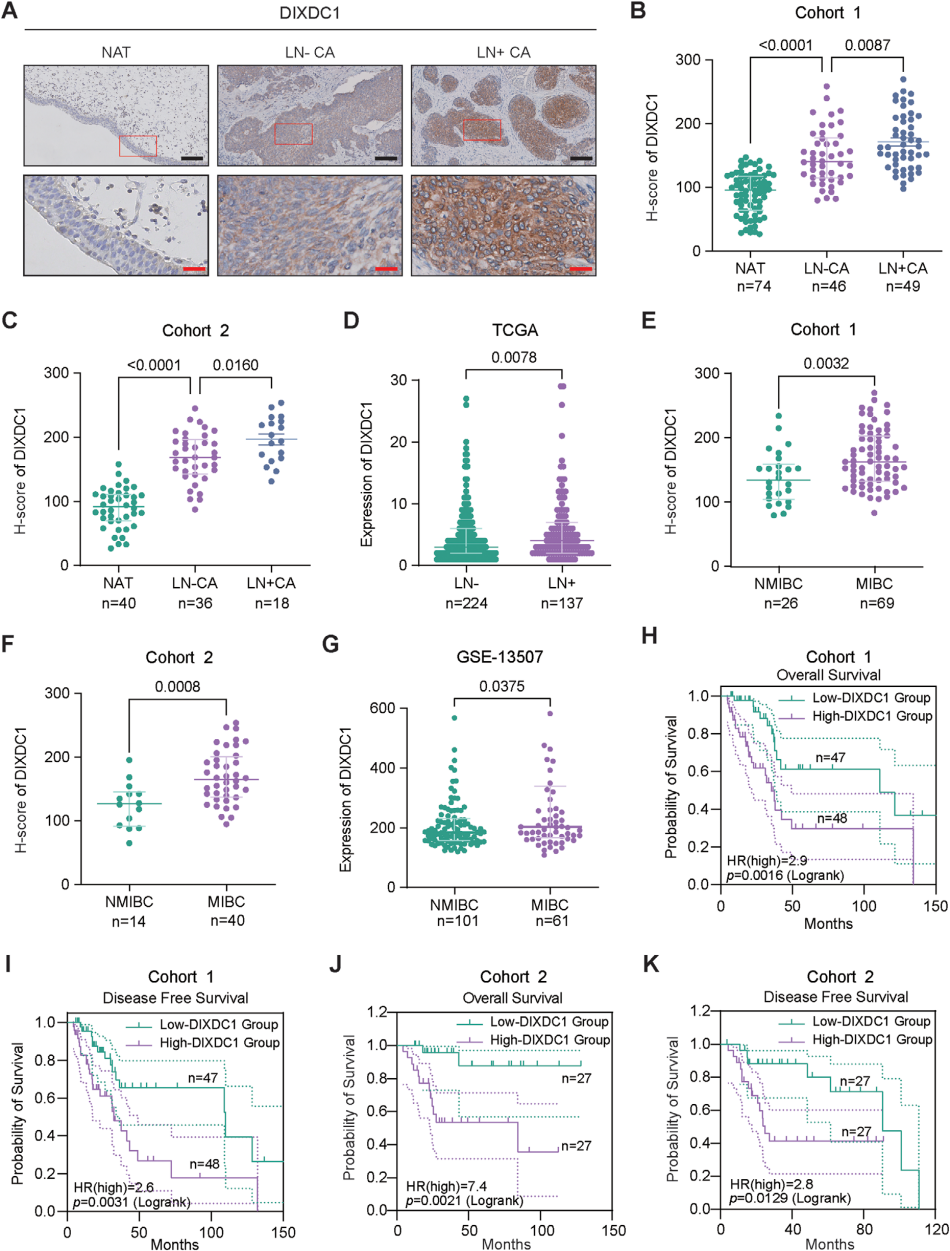
DIXDC1 is overexpressed and associated with poor prognosis in bladder cancer. (A) Representative immunohistochemical (IHC) staining images showing DIXDC1 expression in adjacent normal tissues (NAT), lymph node‐negative bladder cancer tissues (LN^−^ CA), and lymph node‐positive bladder cancer tissues (LN^+^ CA), with high‐magnification images of the red‐boxed regions shown below. Scale bar: 100 µm (black), 20 µm (red). (B, C) Quantitative H‐score analysis of DIXDC1 protein expression in NAT, LN^−^ CA, and LN^+^ CA tissues from Cohort 1 (B) at the Second Affiliated Hospital of Kunming Medical University and Cohort 2 (C) at Sun Yat‐sen Memorial Hospital. (D) Analysis of *DIXDC1* mRNA expression levels in lymph node‐negative and lymph node‐positive bladder cancer samples based on data from The Cancer Genome Atlas (TCGA). (E, F) Comparative analysis of DIXDC1 protein expression between non‐muscle‐invasive bladder cancer (NMIBC) and muscle‐invasive bladder cancer (MIBC) tissues in Cohort 1 (E) and Cohort 2 (F). (G) Analysis of DIXDC1 expression in NMIBC and MIBC samples from the GSE13507 dataset. (H,I) Kaplan‐Meier survival analysis of overall survival (H) and disease‐free survival (I) in bladder cancer patients from Cohort 1, stratified by high and low DIXDC1 expression. (J, K) Kaplan‐Meier survival analysis of overall survival (J) and disease‐free survival (K) in bladder cancer patients from Cohort 2, stratified by high and low DIXDC1 expression. Data are presented as means ± standard deviations (SD). Statistical analyses were performed using ordinary one‐way ANOVA followed by Tukey's multiple‐comparisons test for panels (B, C), the Mann‐Whitney U test for panels (D, G), the unpaired Student's *t*‐test for panels (E, F), and the log‐rank (Mantel‐Cox) test for panels (H–K).

### 2.2 DIXDC1 Promotes the Metastasis‐Related Behaviors of BCa Cells In Vitro and Enhances Lymph Node Metastasis In Vivo

To clarify the functional role of DIXDC1 in BCa cell metastasis, we established DIXDC1‐overexpressing and DIXDC1‐knockdown models in T24 and UM‐UC‐3 cells, respectively, and confirmed the effective regulation of DIXDC1 expression by Western blot and qRT‐PCR (Figure 2A,B and Figure S2A,B). We then systematically evaluated the effect of DIXDC1 on metastasis‐related behaviors in vitro. Consistent results from the wound healing, migration, and invasion assays demonstrated that DIXDC1 overexpression significantly enhanced the migration and invasion capacities of T24 and UM‐UC‐3 cells, whereas DIXDC1 knockdown markedly inhibited the cell migration rate, number of migrated cells, and invasion capacity (Figure 2C–J and Figure S2C), indicating that DIXDC1 significantly promoted the metastasis‐associated phenotypes of BCa cells in vitro.

**FIGURE 2:**
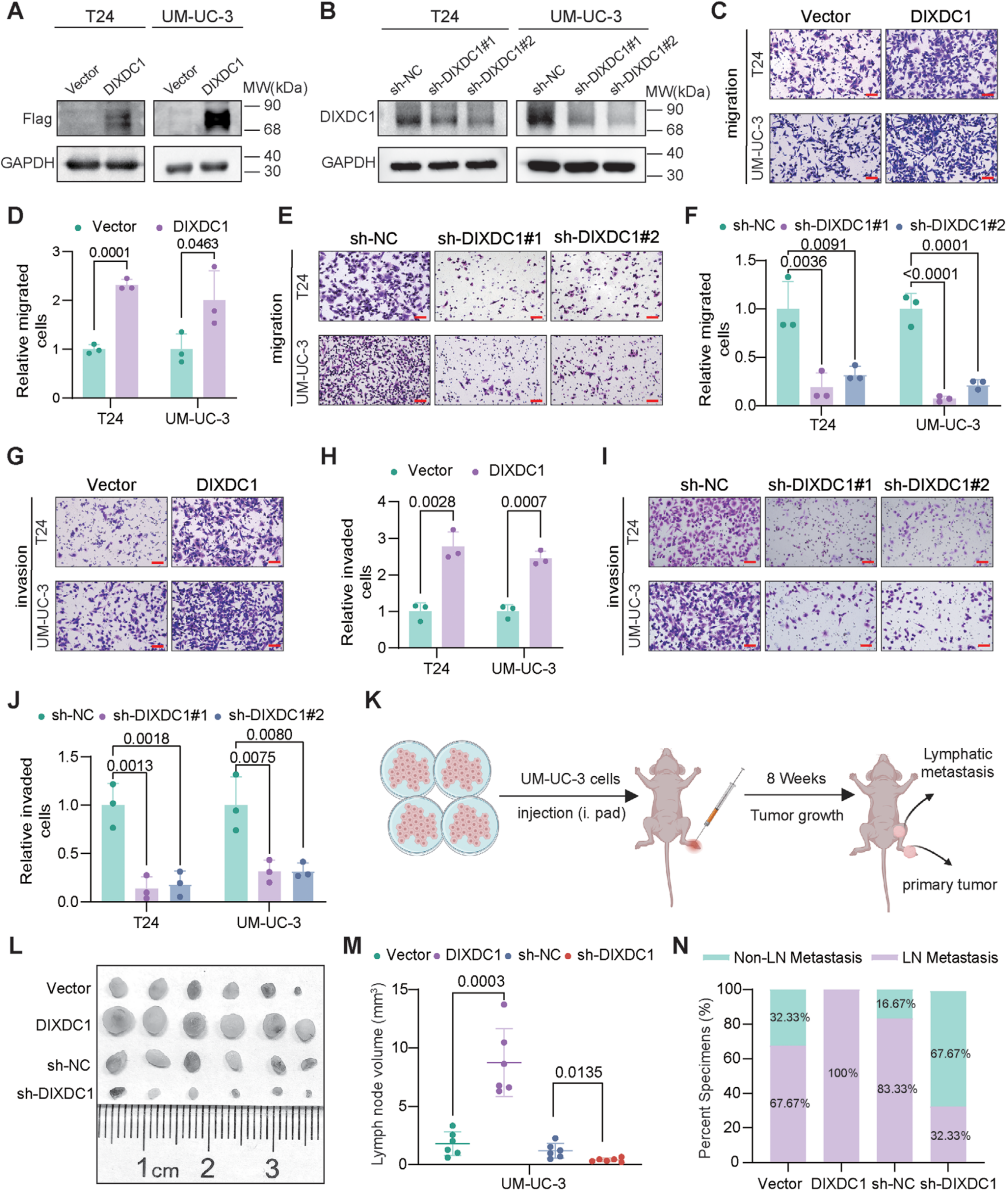
DIXDC1 promotes the metastasis‐related behaviors of bladder cancer cells in vitro and enhances lymph node metastasis in vivo. (A) Verification of DIXDC1 overexpression in T24 and UM‐UC‐3 cells transfected with either an empty vector or a DIXDC1 expression construct by Western blot. (B) Assessment of DIXDC1 protein expression in T24 and UM‐UC‐3 cells with DIXDC1 silencing, determined by Western blot. (C, D) Representative images (C) and quantitative analysis of migrated cell counts (D) from Transwell migration assays in T24 and UM‐UC‐3 cells overexpressing DIXDC1. (E, F) Representative images (E) and quantitative analysis of migrated cell counts (F) in DIXDC1 silencing. (G, H) Representative images (G) and quantitative analysis of invaded cell counts (H) in overexpressing DIXDC1. (I, J) Representative images (I) and quantitative analysis of invaded cell counts (J) in DIXDC1 silencing. Scale bar: 200 µm (red). (K) Schematic diagram illustrating the experimental workflow for establishing a murine plantar footpad injection/lymph node metastasis model with popliteal lymph node metastasis using UM‐UC‐3 cells. (L) Representative gross anatomical images of corresponding popliteal lymph nodes in UM‐UC‐3 cells stably expressing Vector, DIXDC1, sh‐NC, or sh‐DIXDC1. (M) Quantitative analysis of popliteal lymph node volumes across different experimental groups. (N) Quantification of the proportion of specimens with or without lymph node metastasis in each experimental group. Data are expressed as means ± standard deviations (SD). Statistical analyses were performed using unpaired Student's *t*‐tests for panels (D, H, M), and one‐way ANOVA followed by Dunnett's multiple‐comparisons test for panels (F, J).

Furthermore, the involvement of DIXDC1 in lymph node metastasis was investigated in vivo using a footpad injection model. UM‐UC‐3 cells with stable DIXDC1 overexpression or knockdown were inoculated into the footpads of nude mice to establish a popliteal lymph node metastasis model (Figure 2K). Relative to control animals, enforced DIXDC1 expression resulted in a marked enlargement of popliteal lymph nodes, whereas suppression of DIXDC1 led to a significant reduction in lymph node size (Figure 2L,M). In parallel, H&E staining and GFP immunohistochemical analysis showed increased lymph node metastatic involvement in the DIXDC1‐overexpressing group, whereas DIXDC1 silencing substantially reduced metastatic incidence and GFP‐positive tumor burden (Figure 2N and Figure S2D–F). Taken together, these findings demonstrate that DIXDC1 facilitates the migratory and invasive behavior of bladder cancer cells in vitro and drives lymph node dissemination in vivo, highlighting its pivotal pro‐metastatic function during bladder cancer progression.

### 2.3 DIXDC1 Binds and Stabilizes DLAT by Inhibiting Its Ubiquitin‐Mediated Degradation

Given that DIXDC1 is a cytoplasmic scaffolding protein whose biological functions are commonly mediated through protein interactions, we first sought to identify DIXDC1 interacting proteins that may mediate its pro‐metastatic function in BCa cells. Co‐immunoprecipitation followed by silver staining revealed a distinct differential band at approximately 55–70 kDa in T24 and UM‐UC‐3 cells, which was identified as DLAT by mass spectrometry (Figure 3A). DLAT is the E2 subunit of the pyruvate dehydrogenase complex and a core cuproptosis‐associated target [11, 31]. In silico structural prediction and molecular docking supported a potential DIXDC1‐DLAT interaction (Figure 3B), which was further confirmed by endogenous Co‐IP in T24 and UM‐UC‐3 cells (Figure 3C). Confocal immunofluorescence microscopy also revealed cytoplasmic colocalization of DIXDC1 and DLAT, supported by Pearson correlation and line scan analyses in T24 cells (Figure 3D and Figure S3A,B).

**FIGURE 3:**
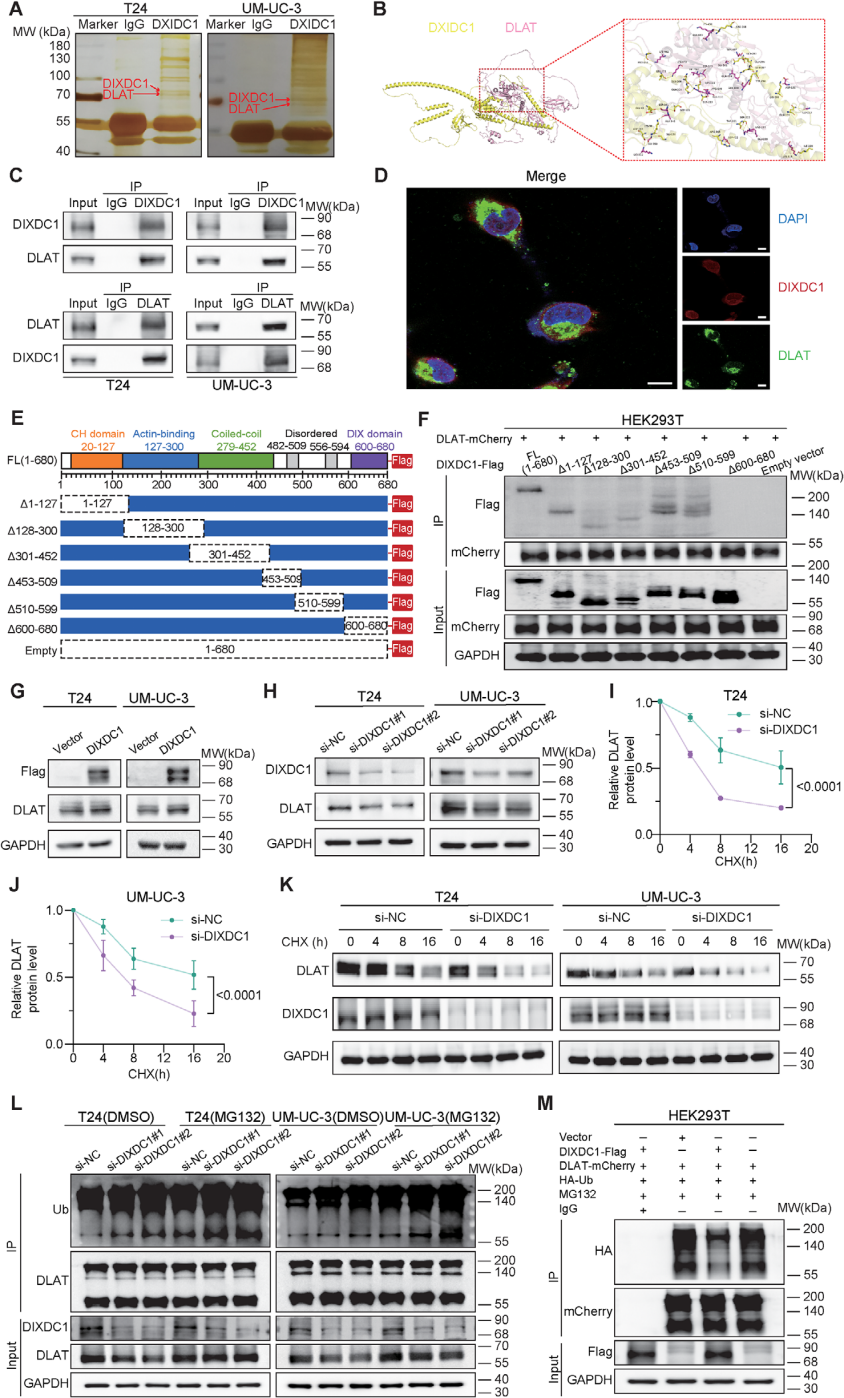
DIXDC1 binds and stabilizes DLAT by inhibiting its ubiquitin‐mediated degradation. (A) Silver staining analysis following co‐immunoprecipitation of endogenous DIXDC1 in T24 and UM‐UC‐3 cells, with DIXDC1‐interacting proteins identified by mass spectrometry; the red arrows indicate the bands corresponding to DIXDC1 and DLAT. (B) In silico prediction of the DIXDC1‐DLAT interaction and their potential binding interface. Structural domains of DIXDC1 (yellow) and DLAT (pink) were retrieved from the Protein Data Bank (PDB). Rigid protein‐protein docking was performed using the Gram‐X web server, and the predicted DIXDC1‐DLAT interaction and binding interface were visualized using PyMOL. (C) Co‐immunoprecipitation assays confirming the endogenous protein‐protein interaction between DIXDC1 and DLAT in T24 and UM‐UC‐3 cells. (D) Confocal immunofluorescence microscopy showing the subcellular colocalization of DIXDC1 and DLAT. Scale bar: 10 µm (white). (E, F) Schematic representation of DIXDC1 deletion mutants used to evaluate the contribution of different DIXDC1 regions to DLAT binding (E), and co‐immunoprecipitation analysis of DLAT‐mCherry with these constructs to assess region‐dependent changes in the DIXDC1‐DLAT interaction (F). The deletion boundaries and reading frames of all DIXDC1 constructs were confirmed by colony PCR and Sanger sequencing before Co‐IP assays. (G) Western blot analysis of DLAT protein expression in T24 and UM‐UC‐3 cells with DIXDC1 overexpression. (H) Western blot analysis of DLAT protein expression in T24 and UM‐UC‐3 cells with DIXDC1 silencing. (I–K) CHX chase assays of DLAT protein stability in T24 and UM‐UC‐3 cells after DIXDC1 knockdown. Cells were treated with cycloheximide (CHX; 20 µg/mL) for 0, 4, 8, and 16 h, with quantification of relative DLAT protein levels in T24 (I) and UM‐UC‐3 (J), and representative immunoblotting results (K). (L) Ubiquitination levels of endogenous DLAT detected by DLAT immunoprecipitation followed by anti‐ubiquitin immunoblotting in T24 and UM‐UC‐3 cells treated with DMSO or MG132 (10 µm, 12 h). Input samples show DIXDC1 knockdown efficiency, total DLAT protein levels, and GAPDH loading controls; IP‐DLAT indicates comparable DLAT immunoprecipitation efficiency across groups. (M) HEK293T cells were co‐transfected with DIXDC1‐Flag, DLAT‐mCherry, and HA‐Ub, followed by MG132 treatment (10 µm, 12 h); DLAT ubiquitination levels were measured by immunoprecipitation using anti‐mCherry. All data are presented as means ± standard deviations (SD). Statistical analyses were performed using two‐way ANOVA followed by Šídák's multiple‐comparisons test for panels (I, J).

A series of DIXDC1 deletion mutants was then generated to map regions involved in DLAT binding. Co‐IP analysis with DLAT‐mCherry revealed region‐dependent changes in DLAT binding, with deletion of the 600 to 680 aa region, corresponding to the C‐terminal DIX domain of DIXDC1, causing the most prominent reduction in the interaction between DIXDC1 and DLAT. Given that the DIX domain is a conserved module involved in scaffold‐mediated protein interactions, this result suggests that the C‐terminal DIX domain contributes substantially to DLAT binding. Deletion of other regions also weakened DLAT binding to varying degrees, suggesting that DIXDC1 may interact with DLAT through multiple regions (Figure 3E,F). DIXDC1 overexpression increased DLAT protein abundance, whereas DIXDC1 silencing reduced DLAT protein levels without altering DLAT mRNA expression (Figure 3G,H and Figure S3C,D). CHX chase assays further showed that DLAT protein declined more rapidly in DIXDC1‐knockdown cells than in control cells, indicating that DIXDC1 depletion shortened the apparent half‐life of DLAT protein and accelerated its degradation (Figure 3I–K). Consistently, DIXDC1 knockdown increased endogenous DLAT polyubiquitination after MG132 treatment, whereas DIXDC1 overexpression reduced DLAT‐mCherry ubiquitination in HEK293T cells expressing HA‐Ub (Figure 3L,M). These findings indicate that DIXDC1 stabilizes DLAT by suppressing ubiquitin‐mediated proteasomal degradation.

We further assessed whether DLAT contributes to DIXDC1‐driven metastatic phenotypes. DLAT knockdown in DIXDC1‐overexpressing T24 and UM‐UC‐3 cells attenuated the enhanced migratory and invasive capacities induced by DIXDC1 overexpression, as shown by Transwell migration and invasion assays (Figure S3E–H). Wound‐healing assays consistently showed reduced wound closure ability after DLAT depletion in both cell lines (Figure S3I–K). These results identify DLAT as a DIXDC1‐interacting protein stabilized by DIXDC1 and support the contribution of the DIXDC1‐DLAT axis to the metastatic behavior of BCa cells.

### 2.4 DLAT is Highly Expressed in BCa and Promotes Aggressive Tumor Progression

To characterize DLAT expression patterns and their clinical significance in bladder cancer, clinical specimens from two independent cohorts, together with data from the TCGA database, were comprehensively analyzed. Analysis of protein expression revealed markedly elevated DLAT levels in bladder cancer tissues relative to paired adjacent normal tissues, with further enrichment observed in lymph node‐positive, muscle‐invasive, and distant metastatic tumors. Consistently, TCGA transcriptomic data demonstrated increased DLAT mRNA abundance in high‐grade bladder cancer samples (Figure S4A–G). In addition, higher DLAT expression was associated with significantly shorter overall and disease‐free survival in both clinical cohorts (Figure S4H–K). Collectively, these findings indicate that DLAT is aberrantly upregulated in bladder cancer and that elevated DLAT expression is closely linked to tumor progression and unfavorable clinical outcomes.

Given the significant clinical relevance of DLAT in BCa, we further explored its functional role in regulating malignant phenotypes in vitro. After DLAT was knocked down in T24 and UM‐UC‐3 cells and the knockdown efficiency was confirmed (Figure S5A,B), functional assays demonstrated that DLAT depletion significantly inhibited the migration and invasion capacities of BCa cells. This was reflected by a marked decrease in the wound healing rate in the wound healing assays (Figure S5C) and a significant reduction in the number of migrated and invaded cells in Transwell assays (Figure S5D–G). Additionally, CCK‐8 and colony formation assays revealed that DLAT knockdown significantly suppressed the proliferation and colony‐forming abilities of T24 and UM‐UC‐3 cells (Figure S5H–K). These findings indicate that DLAT significantly promotes the migration, invasion, and proliferation of BCa cells in vitro, supporting its oncogenic role in BCa progression.

### 2.5 DIXDC1 Regulates the Expression and mRNA Stability of NEK7 and CCNE2 Via DLAT

To explore downstream mediators of the DIXDC1‐DLAT axis, we performed transcriptome sequencing in DIXDC1‐knockdown T24 and UM‐UC‐3 cells. Differential expression and volcano plot analyses identified a group of genes significantly downregulated after DIXDC1 silencing in both cell lines (Figure 4A,B). Among these downregulated genes, candidates previously reported to be associated with tumor progression, cell cycle regulation, or metastatic phenotypes were selected through literature screening and then subjected to qRT‐PCR validation in parallel DIXDC1‐knockdown and DLAT‐knockdown models. Intersection analysis identified CCNE2 and NEK7 as the candidate genes most consistently reduced under both depletion conditions in T24 and UM‐UC‐3 cells, supporting their selection as shared downstream candidates affected by DIXDC1 and DLAT (Figure 4C–F). At the protein level, DIXDC1 overexpression increased, whereas DIXDC1 knockdown reduced, NEK7 and CCNE2 expression in T24 and UM‐UC‐3 cells (Figure 4G,H). In DIXDC1‐overexpressing cells, subsequent DLAT silencing attenuated the upregulation of CCNE2 and NEK7 at both the mRNA and protein levels (Figure 4I–K), indicating that DIXDC1 regulates their expression through DLAT.

**FIGURE 4:**
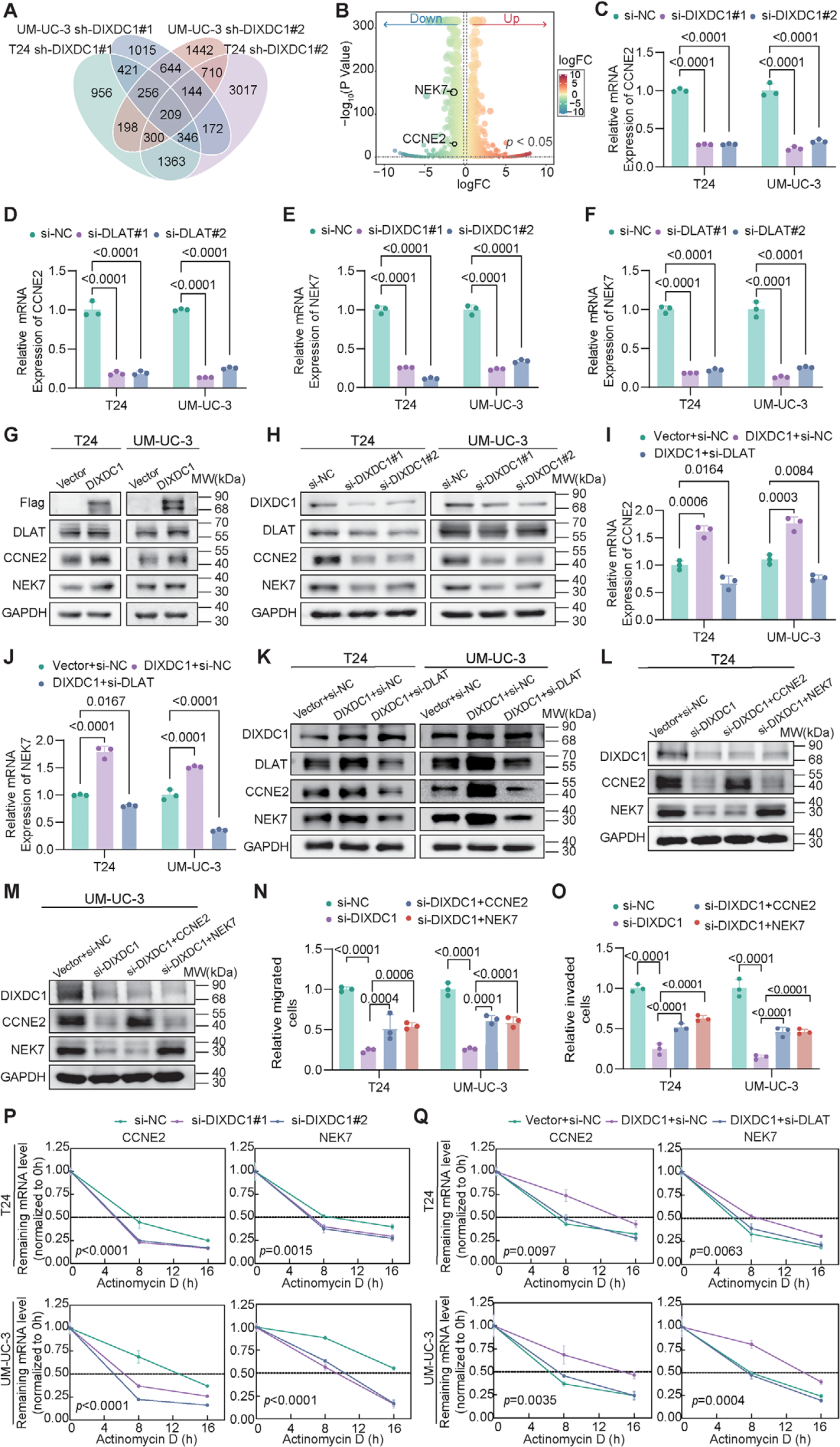
DIXDC1 regulates the expression and mRNA stability of *NEK7* and *CCNE2* via DLAT. (A) Transcriptome sequencing analysis of T24 and UM‐UC‐3 cells with DIXDC1 knockdown to identify differentially expressed genes (DEGs), with DEG criteria defined as |log_2_ fold change| ≥ 0.585, corresponding to a 1.5‐fold change, and adjusted *p* value (FDR) < 0.05. (B) Volcano plot of DEGs following DIXDC1 knockdown, with the downregulated candidate genes CCNE2 and NEK7 highlighted. (C–F) qRT‐PCR quantification of relative mRNA expression of *CCNE2* and *NEK7* in T24 and UM‐UC‐3 cells. Transcript levels of CCNE2 and NEK7 following transfection with si‐NC or two separate DIXDC1‐targeting siRNAs (C, E); transcript levels of *CCNE2* and *NEK7* following transfection with si‐NC or two separate DLAT‐targeting siRNAs (D, F). (G, H) Western blot analysis was conducted to determine the protein levels of DIXDC1, DLAT, NEK7, and CCNE2 in T24 and UM‐UC‐3 cells under conditions of DIXDC1 overexpression (G) or knockdown (H). (I, J) qRT‐PCR quantification of *CCNE2* (I) and *NEK7* (J) mRNA expression in T24 and UM‐UC‐3 cells with DIXDC1 overexpression following subsequent DLAT silencing. (K) Western blot analysis of DLAT, NEK7, and CCNE2 protein expression in T24 and UM‐UC‐3 cells with DIXDC1 overexpression and subsequent DLAT silencing. (L, M) Western blot analysis of NEK7 and CCNE2 expression in T24 (L) and UM‐UC‐3 (M) cells following DIXDC1 knockdown. (N, O) Transwell migration (N) and invasion (O) assays showing that NEK7 or CCNE2 overexpression partially attenuates the suppressive effects of DIXDC1 knockdown on the migratory and invasive abilities of T24 and UM‐UC‐3 cells. (P) T24 and UM‐UC‐3 cells with DIXDC1 knockdown were treated with actinomycin D (10 µg/mL), and *CCNE2* and *NEK7* mRNA expression levels were examined at different time points. (Q) T24 and UM‐UC‐3 cells with DIXDC1 overexpression and subsequent DLAT silencing were treated with actinomycin D (10 µg/mL), and *CCNE2* and *NEK7* mRNA expression levels were assessed at various time points. All data are presented as means ± standard deviations (SD). Statistical analyses were performed using one‐way ANOVA followed by Dunnett's multiple‐comparisons test for panels (C–F), (I, J, N, O), and two‐way ANOVA followed by Dunnett's multiple‐comparisons test for panels (P, Q).

Considering that previous studies have implicated NEK7 and CCNE2 not only in cell‐cycle regulation but also in tumor migration, invasion, and metastatic progression [23, 24, 25], we next assessed their functional relevance in DIXDC1‐regulated metastatic phenotypes. NEK7 or CCNE2 was overexpressed in DIXDC1‐knockdown cells, and western blotting verified the overexpression of NEK7 or CCNE2 in these rescue settings (Figure 4L,M). Transwell assays showed that NEK7 or CCNE2 overexpression partially rescued the inhibitory effects of DIXDC1 knockdown on migration and invasion (Figure 4N,O and Figure S6A,B). These findings support that NEK7 and CCNE2 contribute to DIXDC1‐regulated migratory and invasive phenotypes. Finally, actinomycin D chase assays showed that DIXDC1 knockdown accelerated *CCNE2* and *NEK7* mRNA decay, whereas DIXDC1 overexpression prolonged their transcript stability; this effect was weakened by subsequent DLAT knockdown (Figure 4P,Q). These findings indicate that DIXDC1 maintains *NEK7* and *CCNE2* expression via DLAT‐mediated enhancement of mRNA stability, thereby supporting the migratory and invasive capacities of BCa cells.

### 2.6 DIXDC1 Promotes Bladder Cancer Cell Proliferation In Vitro

During the literature‐based prioritization of candidate downstream genes, NEK7 and CCNE2 were noted to be closely associated with cell‐cycle regulation and proliferative progression. We therefore examined whether DIXDC1 affects BCa cell proliferation. CCK‐8 and colony formation assays showed that DIXDC1 overexpression enhanced cell growth and clonogenic capacity, whereas DIXDC1 knockdown suppressed these proliferative phenotypes in T24 and UM‐UC‐3 cells (Figure 5A–H). Consistently, flow cytometry analysis showed that DIXDC1 knockdown induced G0/G1‐phase accumulation, whereas DIXDC1 overexpression promoted cell‐cycle progression (Figures S7A–G and S8A–D). These findings indicate that DIXDC1 enhances the proliferative capacity of BCa cells.

**FIGURE 5:**
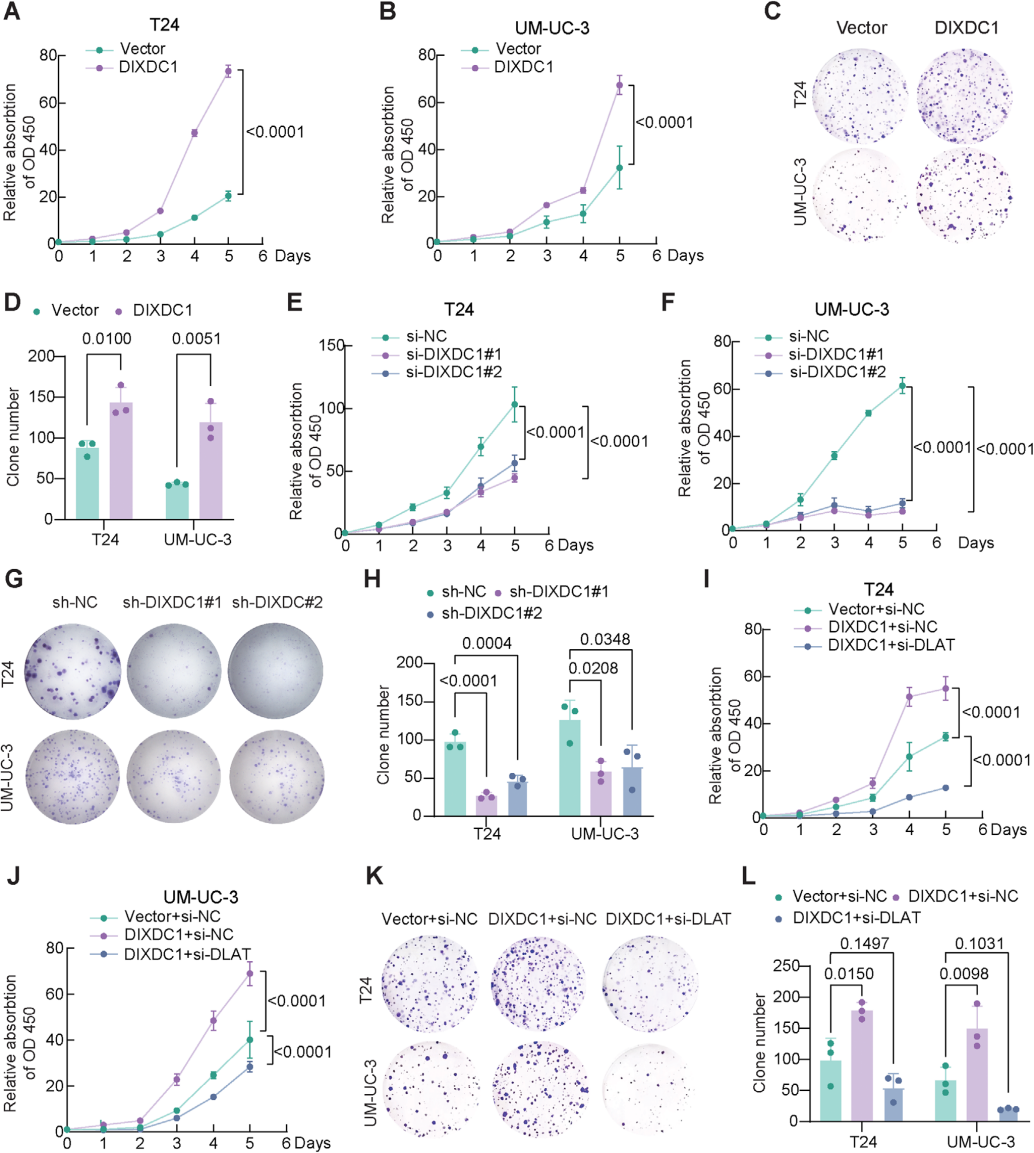
DIXDC1 promotes bladder cancer cell proliferation in vitro. (A, B) Cell viability assays at multiple time points using CCK‐8 and growth curve generation in DIXDC1‐overexpressing T24 (A) and UM‐UC‐3 (B) cells. (C, D) Colony formation assay in DIXDC1‐overexpressing T24 and UM‐UC‐3 cells, with representative colony images (C) and corresponding quantitative analysis of colony formation efficiency (D). (E, F) Cell viability measurement at different time points by CCK‐8 and growth curve plotting in T24 (E) and UM‐UC‐3 (F) cells with DIXDC1 knockdown. (G, H) Colony formation assay in T24 and UM‐UC‐3 cells with DIXDC1 knockdown, with representative colony images (G) and corresponding quantitative analysis of colony numbers (H). (I, J) In T24 (I) and UM‐UC‐3 (J) cells with stable overexpression of DIXDC1, DLAT was further knocked down, and cell growth curves were evaluated using the CCK‐8 assay. (K, L) Colony formation assay under the indicated experimental conditions, with representative colony images (K) and corresponding quantitative analysis of colony numbers (L). Data are presented as means ± standard deviations (SD). Statistical analyses were performed using two‐way ANOVA followed by Šídák's multiple‐comparisons test for panels (A, B), two‐way ANOVA followed by Dunnett's multiple‐comparisons test for panels (E, F, I, J), unpaired Student's *t*‐test for panel (D), and one‐way ANOVA followed by Dunnett's multiple‐comparisons test for panels (H, L).

Given that DLAT was identified as a DIXDC1‐stabilized downstream effector, we further examined whether DLAT depletion affected DIXDC1‐driven proliferation. In DIXDC1‐overexpressing T24 and UM‐UC‐3 cells, subsequent DLAT knockdown reduced DIXDC1‐induced cell growth and clonogenic capacity, as assessed by CCK‐8 and colony formation assays (Figure 5I–L). These results indicate that DIXDC1 promotes BCa cell proliferation, whereas DLAT depletion weakens the proliferative advantage induced by DIXDC1 overexpression.

### 2.7 DIXDC1 Promotes Resistance to Copper‐Induced Cuproptosis in Bladder Cancer

Given that DLAT is a core cuproptosis‐associated target [26] directly regulated by DIXDC1, we next examined whether DIXDC1 affects the sensitivity of BCa cells to cuproptosis. Copper ion levels were higher in BCa tissues than in paired adjacent normal tissues (Figure 6A), and bladder cancer cell lines exhibited relatively higher copper ion levels than the normal uroepithelial cell line SV‐HUC‐1 (Figure 6B). These data mainly reflect the elevated copper metabolic background of BCa tissues and cells, providing a biological context for further investigation of cuproptosis sensitivity in BCa. CCK‐8 assays showed dose‐dependent sensitivity of T24 and UM‐UC‐3 cells to elesclomol combined with Cu(II) (ES‐Cu), allowing IC_50_ calculation in both cell lines (Figure S7H,I). DIXDC1 knockdown decreased the IC_50_ of ES‐Cu, whereas DIXDC1 overexpression increased ES‐Cu IC_50_ values in T24 and UM‐UC‐3 cells (Figure 6C,D), indicating that DIXDC1 reduces BCa cell sensitivity to ES‐Cu‐induced cytotoxicity.

**FIGURE 6:**
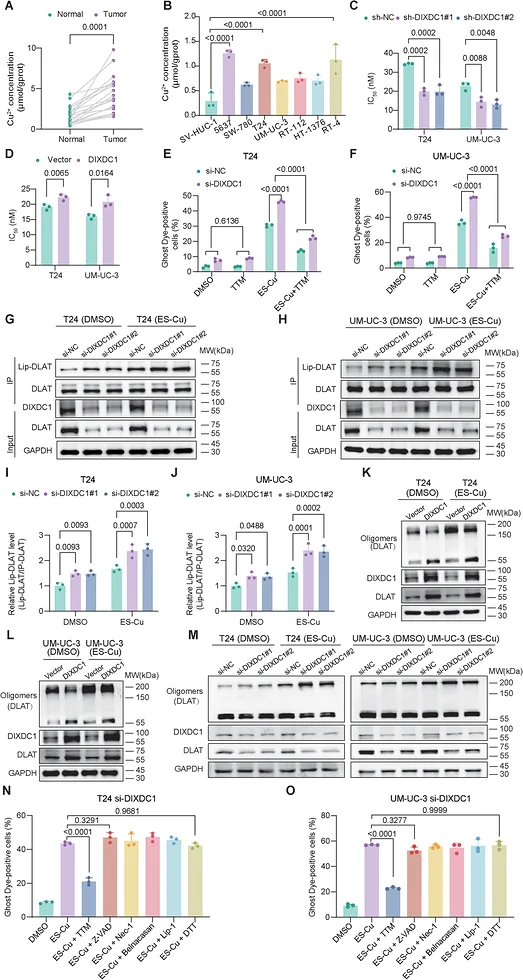
DIXDC1 promotes resistance to copper‐induced cuproptosis in bladder cancer. (A) Measurement of copper ion concentration in paired bladder cancer tissues and their corresponding adjacent normal tissues using a copper ion detection kit, with each connecting line representing an individual patient. (B) The copper ion content was measured in the normal uroepithelial cell line SV‐HUC‐1 and bladder cancer cell lines (5637, SW‐780, T24, UM‐UC‐3, RT‐112, HT‐1376, RT‐4) using a commercial copper ion detection kit. (C) Determination of the half‐maximal inhibitory concentration (IC_50_) of the copper ionophore Elesclomol combined with Cu (II) (ES‐Cu) in T24 and UM‐UC‐3 cells with DIXDC1 knockdown. (D) Measurement of ES‐Cu IC_50_ in DIXDC1‐overexpressing T24 and UM‐UC‐3 cells. (E, F) Ghost dye staining followed by flow cytometry analysis of cell death in T24 (E) and UM‐UC‐3 (F) cells under the indicated treatments. Quantification of Ghost dye–positive cells is shown in control and DIXDC1 knockdown cells treated with DMSO, TTM, ES‐Cu, or ES‐Cu+TTM. (G, H) Co‐immunoprecipitation analysis of lipoylated DLAT (Lip‐DLAT) and total DLAT in T24 (G) and UM‐UC‐3 (H) cells under DMSO or ES‐Cu treatment with or without DIXDC1 knockdown. Input controls show DIXDC1 and DLAT expression levels. (I, J) Densitometric quantification of Lip‐DLAT normalized to immunoprecipitated DLAT in T24 (I) and UM‐UC‐3 (J) cells. For each cell line, values were further normalized to the mean value of the DMSO‐treated si‐NC group. (K–M) DLAT oligomerization analysis under DIXDC1 overexpression (K and L) or knockdown (M) in T24 and UM‐UC‐3 cells treated with DMSO or ES‐Cu using non‐reducing western blotting. (N, O) Ghost Dye staining followed by flow cytometry analysis of cell death in T24 (N) and UM‐UC‐3 (O) cells under DIXDC1 knockdown. Cells were treated with ES‐Cu alone or in combination with different cell death inhibitors, including TTM, Z‐VAD‐FMK, Nec‐1, Belnacasan (VX‐765), Lip‐1, and DTT. Quantification of Ghost Dye‐positive cells is shown. Data are presented as means ± standard deviations (SD). Statistical analyses were performed using paired Student's *t*‐test for panel (A), unpaired Student's *t*‐test for panel (D), one‐way ANOVA with Dunnett's multiple‐comparisons test for panels (B, C, N, O), two‐way ANOVA with Tukey's or Šídák's multiple‐comparisons test for panels (E, F), and two‐way ANOVA with Dunnett's multiple‐comparisons test for panels (I, J).

Ghost Dye flow cytometry further confirmed that DIXDC1 knockdown enhanced ES‐Cu‐induced cell death in both T24 and UM‐UC‐3 cells, whereas co‐treatment with the copper chelator TTM markedly attenuated this effect (Figure 6E,F and Figure S8E–G). We next examined DLAT‐related cuproptosis events under ES‐Cu treatment. Co‐immunoprecipitation analysis showed that DIXDC1 knockdown increased lipoylated DLAT levels following ES‐Cu exposure, and densitometric quantification of Lip‐DLAT normalized to immunoprecipitated DLAT further confirmed this increase in both T24 and UM‐UC‐3 cells (Figure 6G–J). Consistently, non‐reducing western blot analysis showed that DIXDC1 overexpression reduced DLAT oligomerization, whereas DIXDC1 knockdown increased DLAT oligomer formation under ES‐Cu treatment (Figure 6K–M).

To further distinguish cuproptosis from other cell death modalities, DIXDC1‐knockdown cells were treated with ES‐Cu in combination with different cell death inhibitors or redox intervention reagents. TTM substantially reduced ES‐Cu‐induced cell death, whereas the apoptosis inhibitor Z‐VAD‐FMK, necroptosis inhibitor Nec‐1, pyroptosis inhibitor Belnacasan (VX‐765), ferroptosis inhibitor Lip‐1, and the reducing agent DTT failed to significantly suppress ES‐Cu‐induced cell death (Figure 6N,O). These findings indicate that DIXDC1 enhances BCa cell resistance to cuproptosis, accompanied by reduced DLAT lipoylation and oligomerization under ES‐Cu treatment.

### 2.8 DIXDC1 Modulates the Growth of BCa Cells and Resistance to Cuproptosis In Vivo

To evaluate the role of DIXDC1 in BCa growth and response to ES‐Cu treatment in vivo, subcutaneous xenograft models were established using UM‐UC‐3 cells with stable DIXDC1 overexpression or knockdown and their corresponding control cells. After tumor formation, mice were treated with PBS or ES‐Cu (Figure 7A). DIXDC1 overexpression promoted tumor growth and weakened the antitumor effect of ES‐Cu, whereas DIXDC1 knockdown suppressed tumor growth and enhanced the response to ES‐Cu, as reflected by changes in tumor volume, gross morphology, and final tumor weight (Figure 7B–D,G–I). Histological analyses further indicated that DIXDC1 overexpression promoted tumor cell proliferation and attenuated ES‐Cu‐associated tumor cell death, whereas DIXDC1 knockdown produced the opposite effects (Figure 7E,F,J,K and Figures S9A,B and S10A,B).

**FIGURE 7:**
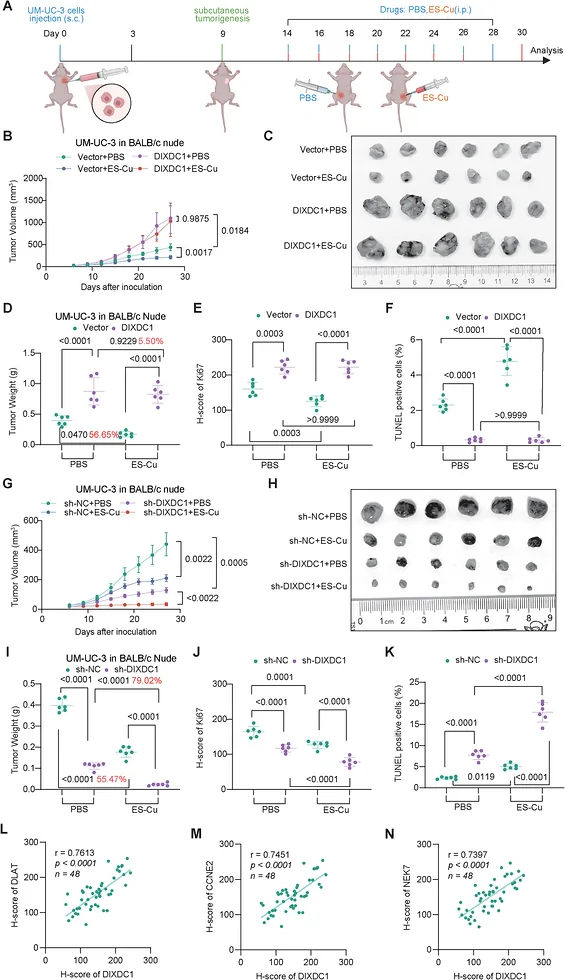
DIXDC1 modulates the growth of bladder cancer cells and resistance to cuproptosis in vivo. (A) Schematic illustration of experimental design. Subcutaneous xenograft models were established in nude mice by inoculating UM‐UC‐3 cells with stable DIXDC1 overexpression or knockdown (and their respective controls). After tumor formation, mice were treated with PBS or ES‐Cu (6 mg/kg, intraperitoneal injection, every 3 days). (B–F) Tumor volume (B), gross morphology (C), final tumor weight (D), Ki67 H‐scores (E), and TUNEL positivity (F) were assessed in xenografts derived from UM‐UC‐3 cells with stable DIXDC1 overexpression. (G–K) Corresponding analyses for tumor volume (G), gross morphology (H), final tumor weight (I), Ki67 H‐scores (J), and TUNEL positivity (K) were performed in xenografts with stable DIXDC1 knockdown. (L–N) Correlation analysis between DIXDC1 expression and the expression of DLAT (L), CCNE2 (M), and NEK7 (N) in subcutaneous xenograft tumors, based on H‐score quantification. All data are presented as means ± standard deviations (SD). Statistical analyses were performed using two‐way ANOVA followed by Tukey's multiple‐comparisons test for panels (B, D–G, I–K), and Pearson correlation analysis for panels (L, M).

We further examined DIXDC1‐related molecular changes in subcutaneous xenograft tumors. Immunohistochemical staining showed that DLAT, CCNE2, and NEK7 expression changed consistently with DIXDC1 expression across the different experimental groups (Figure S11A). Consistently, correlation analysis based on H‐scores revealed positive correlations between DIXDC1 expression and the expression of DLAT, CCNE2, and NEK7 (Figure 7L–N), further supporting the coordinated regulation of these molecules in vivo. H&E staining of liver and kidney tissues showed no obvious histological abnormalities after ES‐Cu treatment under the conditions used in this study (Figure S12A,B). Collectively, these in vivo findings indicate that DIXDC1 promotes BCa tumor growth and confers resistance to ES‐Cu treatment, accompanied by coordinated changes in DLAT, CCNE2, and NEK7 expression.

### 2.9 DIXDC1 Promotes Bladder Cancer Growth and Lymphatic Metastasis Through DLAT In Vivo

In the orthotopic bladder cancer model, DIXDC1 overexpression markedly increased tumor burden at the experimental endpoint, whereas DIXDC1 knockdown reduced tumor growth, as indicated by bioluminescence imaging (Figure 8A). Endpoint tissue analysis further showed that DIXDC1 overexpression increased bladder tumor size and tumor weight and promoted pelvic lymph node metastasis, while DIXDC1 knockdown inhibited orthotopic tumor growth and lymphatic metastatic burden (Figure 8B–E). Stable UM‐UC‐3 cell models with DIXDC1 overexpression and subsequent DLAT knockdown were then established and validated by western blot analysis (Figure 8F), and DLAT knockdown weakened the tumor growth advantage induced by DIXDC1 overexpression in subcutaneous xenograft models, as reflected by slower tumor growth and lower final tumor weight (Figure 8G–I). These findings indicate that DIXDC1 promotes BCa proliferation and lymphatic metastasis through DLAT.

**FIGURE 8:**
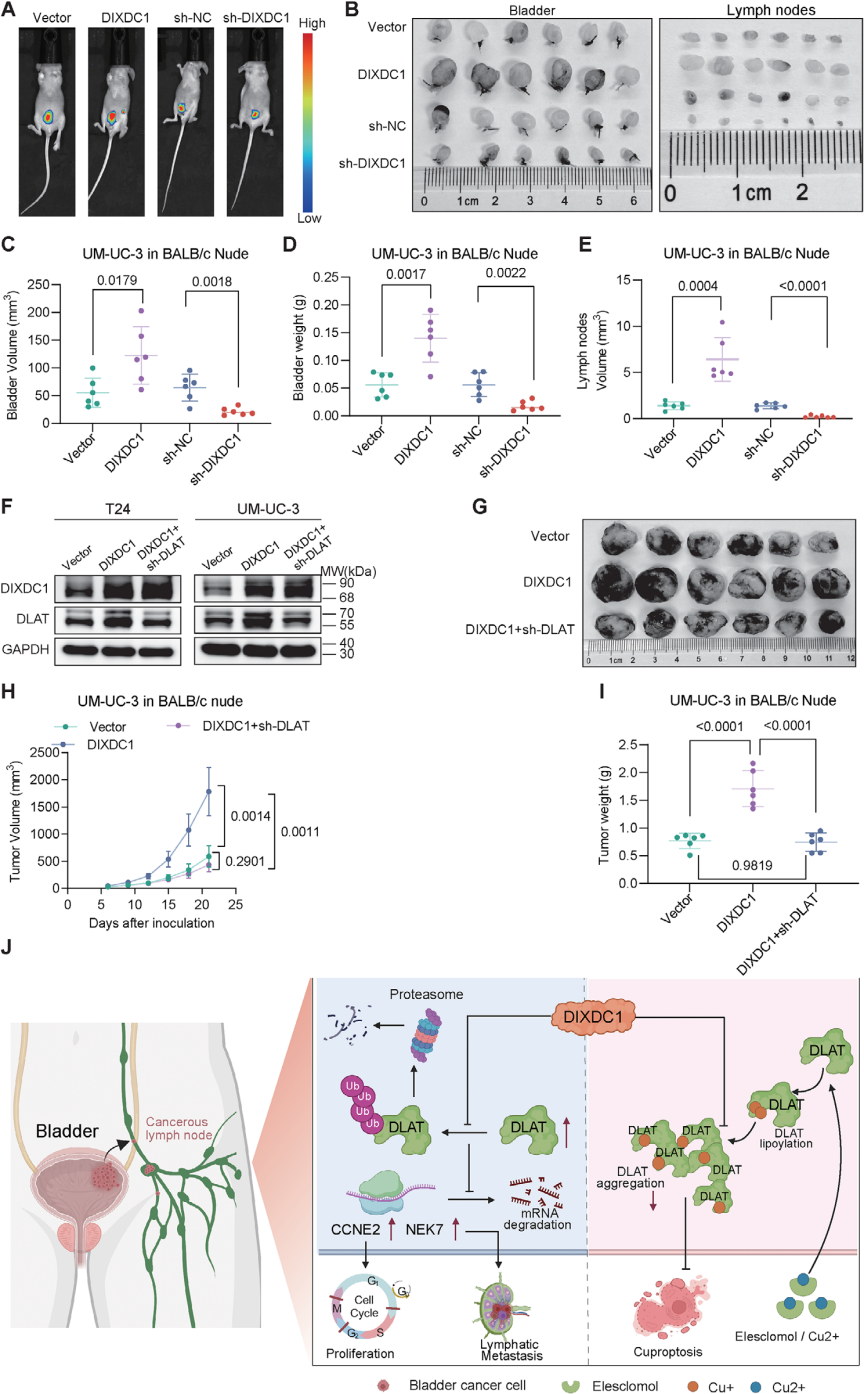
DIXDC1 drives bladder cancer progression and lymphatic metastasis through DLAT in vivo. (A‐F) Orthotopic bladder cancer xenograft models were established by inoculating luciferase‐labeled stable DIXDC1‐overexpressing or DIXDC1‐knockdown human bladder cancer UM‐UC‐3 cells (and their respective control cells) into 4‐5‐week‐old female BALB/c nude mice. (A) Representative in vivo bioluminescence imaging of tumor burden at the experimental endpoint (day 14 post‐inoculation). (B) Representative images of orthotopic bladder tumors and pelvic lymph nodes. (C) Quantification of bladder tumor volume. (D) Measurement of bladder tumor weight. (E) Quantification of pelvic lymph node volume. (F) Western blot analysis confirming DIXDC1 overexpression with subsequent DLAT knockdown in the established rescue stable cell lines. (G–I) Subcutaneous xenograft models were established by inoculating the stable cell lines generated in (F) into 4–5 week old male BALB/c nude mice. (G) Representative images of excised subcutaneous tumors. (H) Tumor growth curves. (I) Final tumor weight at the experimental endpoint. (J) Schematic model illustrating the role of the DIXDC1‐DLAT axis in bladder cancer progression and cuproptosis resistance. DIXDC1 directly interacts with DLAT, exerting a dual regulatory function. On the left, DIXDC1 stabilizes DLAT by inhibiting its ubiquitination and proteasomal degradation. The stabilized DLAT then enhances the mRNA stability of CCNE2 and NEK7, promoting cell cycle progression, proliferation, and lymph node metastasis. On the right, DIXDC1 prevents copper‐induced toxic oligomerization of DLAT, thereby conferring resistance to cuproptosis induced by ES‐Cu. (Created with BioRender.com). Data are presented as means ± standard deviations (SD). Statistical analyses were performed using unpaired Student's *t*‐tests for panels (C–E), two‐way analysis of variance (ANOVA) followed by Tukey's multiple‐comparisons test for panel (H), and one‐way ANOVA followed by Tukey's multiple‐comparisons test for panel (I).

## 3 Discussion

LN metastasis is a major determinant of poor prognosis in BCa and remains a significant challenge in clinical management [27, 28, 29]. In this study, we demonstrate that DIXDC1 is markedly upregulated in lymph node‐metastatic BCa tissues and serves as an independent biomarker for predicting poor patient prognosis. Mechanistically, DIXDC1 stabilizes DLAT protein levels through direct interaction, thereby driving tumor progression. We further confirm that the interaction between DIXDC1 and DLAT has a dual pro‐tumorigenic effect. First, by stabilizing the downstream signaling pathways of CCNE2 and NEK7, it promotes the proliferation and metastasis of BCa cells. Second, it counteracts cuproptosis by limiting DLAT lipoylation and oligomerization under ES‐Cu exposure. These findings establish a direct molecular connection between metastatic signaling and cuproptosis resistance, offering a mechanistic framework for comprehending the progression of aggressive BCa.

DIXDC1, a cytoplasmic scaffolding protein, has been implicated in the initiation and progression of various cancers, primarily through the regulation of classical signaling pathways such as Wnt/β‐catenin and the PI3K‐AKT/AP‐1 pathway [16, 22]. However, its specific role in BCa, particularly in LN metastasis, remains underexplored. In this study, we demonstrate that DIXDC1 promotes BCa cell proliferation, metastasis, and resistance to cuproptosis both in vitro and in vivo. Notably, we identify DLAT as a novel binding partner of DIXDC1. This discovery not only elucidates the mechanism by which DIXDC1 stabilizes DLAT protein levels but also expands its oncogenic function beyond traditional signal transduction to include metabolic regulation. These findings further suggest that DIXDC1 may coordinate Wnt‐related signaling activity with metabolic remodeling during BCa progression, although the precise crosstalk between these processes remains to be further investigated.

Through transcriptome sequencing and intersection‐based validation in DIXDC1 and DLAT knockdown models, NEK7 and CCNE2 emerged as functionally relevant downstream targets whose regulation by DIXDC1 depends on DLAT. We identify an unappreciated mechanism through which the DIXDC1‐DLAT axis augments the post‐transcriptional stability of *NEK7* and *CCNE2* mRNAs, consequently upregulating their expression. Functionally, CCNE2 is a well‐established core cyclin that regulates the G1/S cell cycle transition. Its overexpression activates CDK2, promoting DNA replication, and has been strongly linked to lymph node metastasis and poor prognosis in both BCa and other cancers [30, 31, 32]. Additionally, NEK7, a serine/threonine kinase initially recognized for its role in centrosome dynamics and spindle assembly [11, 12], has been implicated in promoting tumor progression across multiple malignancies through activation of the NLRP3 inflammasome and MAPK signaling pathways [33, 34, 35]. The concomitant regulation of NEK7 and CCNE2 suggests that the DIXDC1‐DLAT axis may affect a shared post‐transcriptional regulatory process rather than independently controlling each transcript. Increasing evidence indicates that metabolic enzymes can exert noncanonical RNA‐regulatory or moonlighting functions beyond their classical metabolic roles. Representative examples include PKM2 and PHGDH, which have been reported to participate in RNA‐associated regulatory programs [36]. In this context, we speculate that DLAT may influence cytoplasmic mRNA stability either through a noncanonical RNA‐regulatory function or indirectly by altering metabolic states or mitochondrial stress responses, which may modulate the activity, localization, or composition of RNA‐binding protein complexes. However, whether DLAT directly binds NEK7 or CCNE2 transcripts remains to be clarified. Collectively, DIXDC1 functions as a key upstream regulator, driving the expression of these critical oncogenes. Consequently, targeting the DIXDC1‐DLAT interaction may help disrupt this progression‐related regulatory network, inhibiting tumor proliferation and metastasis, and may represent a potential therapeutic strategy for BCa.

DLAT, a crucial component of the TCA cycle and pyruvate dehydrogenase complex (PDC), serves as a key bridge between glycolysis and mitochondrial oxidative metabolism, thereby supporting the bioenergetic demands of tumor cells [37, 38]. Accumulating studies suggest that DLAT is overexpressed in several malignancies, such as non‐small cell lung cancer and gastric cancer, and is linked to augmented tumor growth and metastatic potential [39, 40, 41]. Emerging evidence suggests that, in addition to acetylation, DLAT undergoes ubiquitination, highlighting a role for ubiquitin‐dependent regulation in its proteasomal degradation and stability [42, 43]. In this study, we provide the first evidence that DIXDC1 binds directly to DLAT and inhibits its ubiquitination‐mediated degradation, thereby stabilizing DLAT protein levels. Although DLAT mRNA expression may correlate with tumor grade and prognosis in public datasets, this clinical association does not contradict the post‐translational regulation identified in our study. Instead, DLAT expression in BCa may be regulated at multiple levels, including transcriptional, post‐transcriptional, and post‐translational mechanisms. In the specific context of DIXDC1 regulation, our data indicate that DIXDC1 mainly modulates DLAT at the protein stability level rather than at the mRNA level. This discovery introduces a novel regulatory mechanism, underscores the pivotal role of DIXDC1 in tumor metabolism, and offers a new insight into the upstream regulation of DLAT stability.

Recent studies have identified DLAT and copper‐induced toxic oligomerization as key steps in amplifying cuproptosis signaling [44, 45]. Cuproptosis is a form of cell death triggered by excessive copper ions, characterized by the toxic oligomerization of lipoylated TCA cycle enzymes, including DLAT. This process ultimately disrupts mitochondrial protein homeostasis, induces proteotoxic stress, and leads to the loss of Fe–S cluster proteins, thereby inducing cell death [11, 46, 47, 48]. In this study, we demonstrate that DIXDC1 directly interacts with DLAT, thereby limiting DLAT lipoylation and copper‐induced oligomerization under ES‐Cu exposure while stabilizing DLAT protein levels. These effects reduce the sensitivity of BCa cells to ES‐Cu‐induced cuproptosis. This mechanism provides a novel molecular explanation for the metabolic‐survival paradox, wherein metastasis relies on mitochondrial metabolism but remains vulnerable to cuproptosis. Furthermore, this study identifies DIXDC1 as a regulator of DLAT lipoylation and toxic oligomerization under ES‐Cu treatment, suggesting that targeting DIXDC1 may represent a potential therapeutic strategy.

This study also has several limitations. First, DIXDC1 function was mainly investigated using knockdown and overexpression approaches, and DLAT‐dependent rescue experiments were performed using knockdown rather than a complete DLAT knockout background. Future studies using CRISPR/Cas9‐mediated knockout or knock‐in strategies for DIXDC1 and DLAT may provide more direct genetic evidence and further clarify the functional dependence of DIXDC1 on DLAT. Second, although NEK7 and CCNE2 were functionally validated as downstream targets of the DIXDC1‐DLAT axis, other potential downstream effectors and regulatory mechanisms were not comprehensively explored and warrant further investigation. Third, our findings were primarily derived from established BCa cell lines and xenograft models. Further validation in patient‐derived organoids or patient‐derived xenograft models would strengthen the translational relevance of these results. Fourth, the cell death analyses in this study focused on ES‐Cu treatment; therefore, whether DIXDC1 also regulates other forms of regulated cell death in different biological contexts remains to be investigated. In addition, the therapeutic efficacy and safety of copper‐targeting strategies require further systematic evaluation in vivo. Fifth, although multivariate Cox regression analysis supported the potential independent prognostic value of DIXDC1, the current retrospective cohorts, sample size, event number, and follow‐up structure were insufficient to support a robust time‐dependent ROC analysis. Future studies with larger independent cohorts and more complete long‐term follow‐up data will be needed to further evaluate the prognostic predictive performance of DIXDC1.

In summary, we demonstrate that DIXDC1 interacts with DLAT to stabilize DLAT protein levels and regulate its lipoylation and oligomerization, thereby promoting BCa cell proliferation, metastasis, and resistance to cuproptosis (Figure 8J). This mechanistic insight advances our understanding of the oncogenic functions of DIXDC1 and suggests that targeting the DIXDC1‐DLAT axis may confer dual therapeutic benefits by suppressing metastatic progression while restoring sensitivity to cuproptosis. Collectively, these findings highlight the DIXDC1‐DLAT axis as a promising therapeutic vulnerability in advanced BCa.

## 4 Materials and Methods

### 4.1 Patients Tissue Samples

BCa tissues and matched adjacent normal bladder tissues were collected for copper quantification. In addition, paraffin‐embedded specimens obtained between 2022 and 2024 were used for immunohistochemical analyses. All samples were derived from the Second Affiliated Hospital of Kunming Medical University (Cohort 1) and Sun Yat‐sen Memorial Hospital, Sun Yat‐sen University (Cohort 2). Tissue procurement and subsequent analyses were performed in compliance with internationally recognized ethical standards.

### 4.2 Database Analysis

In this study, transcriptomic datasets related to BCa were retrieved from The Cancer Genome Atlas (TCGA) and the Gene Expression Omnibus (GEO) databases. The TCGA BCa (BLCA) cohort was utilized to evaluate the mRNA expression patterns of *DIXDC1* and DLAT and to examine their relationships with clinicopathological features and patient outcomes. Two independent GEO datasets (GSE13507 and GSE87304) were further applied as validation cohorts. All datasets were processed and analyzed following the standardized pipelines and quality‐control guidelines provided by the respective databases.

### 4.3 Immunohistochemistry (IHC) Analysis

Paraffin‐embedded tissue blocks were processed into 4‐µm sections, which were subsequently subjected to deparaffinization and graded rehydration. Antigen epitopes were unmasked by heat‐induced retrieval in Tris‐EDTA buffer (pH 9.0). After blocking of nonspecific interactions, the sections were incubated at 4°C overnight with primary antibodies against DIXDC1, DLAT, NEK7, CCNE2, Ki‐67, and GFP (Table S4). Signal detection was performed using HRP‐linked secondary antibodies, followed by visualization with 3,3′‐diaminobenzidine (DAB) and hematoxylin counterstaining. Whole‐slide images were acquired using a Wanfang pathology digital scanning system.

Immunostaining results were semi‐quantitatively evaluated using the H‐score method, which combines staining intensity with the percentage of positively stained cells for each specimen.

### 4.4 Cell Culture

Human BCa cell lines 5637, SW‐780, T24, UM‐UC‐3, RT‐112, HT‐1376, and RT‐4, the normal human uroepithelial cell line SV‐HUC‐1, and HEK293T cells were cultured in the corresponding complete media according to their requirements. T24 cells were maintained in complete RPMI‐1640 medium, whereas all other cell lines were maintained in complete DMEM. Both cell lines were obtained from the American Type Culture Collection (ATCC, USA). All cell culture experiments were conducted under standardized conditions. To ensure authenticity and exclude cross‐contamination, single‐nucleotide polymorphism (SNP) profiling was performed by IGE Biotechnology Ltd. (Guangzhou, China). All cell lines used in this study were regularly tested for mycoplasma contamination, and no mycoplasma contamination was detected.

### 4.5 RNA Interference

Gene‐specific small interfering RNAs (siRNAs) were purchased from GenePharma (Jiangsu, China). Gene silencing efficiency was validated by qRT‐PCR and immunoblotting, and detailed siRNA sequences are listed in Table S5. For siRNA delivery, transfection complexes were generated using Lipofectamine RNAiMAX (Thermo Fisher Scientific, USA) in Opti‐MEM reduced‐serum medium (Gibco, USA). The complexes were allowed to equilibrate at room temperature for approximately 20 min before being added to cultured cells. After 12–24 h of incubation at 37°C, the transfection medium was replaced with fresh complete growth medium. Cells were subsequently cultured for an additional 24–36 h prior to downstream analyses.

### 4.6 RNA Sequencing Analysis

T24 and UM‐UC‐3 cells were treated with siRNAs targeting DIXDC1 or a non‐targeting negative control for 48 h. Total RNA was extracted with TRIzol reagent (Vazyme, China). Library preparation and next‐generation sequencing were performed by Novogene Co., Ltd. (Beijing, China). Sequencing was carried out using the Illumina HiSeq 6000 system to generate 200‐bp paired‐end reads. Differential gene expression was analyzed using edgeR (v3.42.4). Raw read counts were normalized using the trimmed mean of M‐values (TMM) method implemented in edgeR. P values from differential expression analysis were adjusted using the Benjamini‐Hochberg method to control the false discovery rate. Genes with |log_2_ fold change| ≥ 0.585, corresponding to a 1.5‐fold change, and adjusted *p* value (FDR) < 0.05 were defined as differentially expressed genes. Genes consistently downregulated in both T24 and UM‐UC‐3 cells after DIXDC1 knockdown were selected for subsequent candidate screening and validation. Raw RNA‐seq datasets are available in the Genome Sequence Archive (GSA) with the accession ID HRA016347.

### 4.7 Lentivirus Transduction

Lentiviral vectors encoding shRNAs targeting DIXDC1 or DLAT, together with the corresponding negative control shRNA (sh‐NC) and DIXDC1 overexpression constructs, were designed and generated by IGE Biotechnology (Guangzhou, China). Detailed target information is included in Table S5. Lentiviral packaging and production were carried out by the supplier, and bladder cancer cells were transduced with the indicated viruses with polybrene supplementation (8 µg/mL). Puromycin selection was used to establish stable cell populations, which were subsequently validated by qRT‐PCR together with Western blotting.

### 4.8 RNA Isolation and qRT‐PCR

Total RNA was extracted from cultured cell samples with the Fast RNA Extraction Kit (Squirrel Biotechnology, China) or TRIzol reagent (Vazyme, China), as previously reported [49]. Complementary DNA (cDNA) synthesis was carried out using the HiScript II Q RT SuperMix for qPCR (Vazyme, China). Quantitative real‐time PCR assays were conducted using the ChamQ Universal SYBR qPCR Master Mix (Vazyme, China) and run on a LightCycler 384 real‐time PCR platform (Roche, Basel, Switzerland). Relative mRNA levels were calculated using the 2^−ΔΔCt^ approach, with GAPDH and 18S rRNA used as endogenous reference controls. The primer sets utilized for qRT‐PCR analysis are detailed in Table S6.

### 4.9 Western Blot Assay

Whole‐cell protein extracts were prepared in lysis buffer containing protease and phosphatase inhibitor cocktails. Following protein quantification, samples with matched protein amounts were loaded for SDS‐PAGE separation, after which proteins were transferred onto PVDF membranes. The membranes were conditioned in 5% non‐fat milk at room temperature for 1 h and then exposed to primary antibodies against DIXDC1, DLAT, NEK7, CCNE2, GAPDH, FLAG, mCherry, and HA‐tag (Table S4) during overnight incubation at 4°C. After extensive rinsing with TBST, membranes were reacted with the corresponding secondary antibodies for 1.5 h at room temperature. Signal development was achieved using a chemiluminescent detection platform.

### 4.10 Co‐Immunoprecipitation (Co‐IP) and Mass Spectrometry

Whole‐cell extracts from BCa cells were generated in immunoprecipitation buffer containing protease inhibitors. Co‐immunoprecipitation was performed essentially as previously described [50]. Briefly, clarified lysates were incubated with the indicated primary antibodies or control IgG at 4°C overnight, followed by enrichment of immune complexes using protein A/G magnetic beads (MCE, USA) for 2 h at room temperature. After extensive washing, precipitated proteins were recovered by thermal denaturation in sample buffer and analyzed by Western blotting. For Co‐IP‐MS analysis, immunoprecipitated protein complexes were separated by SDS‐PAGE and visualized by silver staining. Gel bands were excised and subjected to liquid chromatography‐tandem mass spectrometry (LC‐MS/MS) analysis to identify candidate DIXDC1‐interacting proteins. High‐confidence interactors from Co‐IP‐MS are shown in Table S7.

### 4.11 Wound Healing, Migration, and Invasion Assays

Cell motility was evaluated by wound‐healing, Transwell migration, and invasion assays. For wound‐healing experiments, cells were plated in 6‐well plates and cultured until reaching confluence. A straight scratch was created using a sterile 200 µL pipette tip, followed by gentle washing with PBS to remove detached cells. Cells were then maintained in serum‐free medium, and images were captured at the indicated time points. Wound closure was calculated as the percentage decrease in scratch width relative to the initial time point.

Directional cell motility was evaluated using a two‐chamber culture system separated by an 8‐µm‐pore polycarbonate membrane (Corning, USA). Cells (8 × 10^4^) were prepared in 200 µL serum‐deprived medium and placed into the upper chamber. To establish a serum gradient, 700 µL complete medium supplemented with 10% fetal bovine serum was added to the lower chamber. After incubation for 12–24 h, depending on the cell line, cells that traversed the membrane were preserved with paraformaldehyde, visualized by 0.1% crystal violet staining, and quantified by microscopic examination.

For invasion assays, Transwell inserts were precoated with Matrigel (Corning, USA) before cell seeding. Following 12–36 h of incubation, invasive cells were fixed, stained, and quantified using the same procedures as those described for the migration assays.

### 4.12 Footpad Lymph Node Metastasis Model

To establish a lymphatic metastasis model, male BALB/c nude mice (4–5 weeks old, SPF grade) were allocated into four experimental cohorts (Vector, DIXDC1, sh‐NC, and sh‐DIXDC1; *n* = 6 per group). Tumor cells (3 × 10^6^) suspended in 50 µL phosphate‐buffered saline were inoculated into the right hind footpad of each mouse. During the course of the experiment, local tumor development and enlargement of the draining popliteal lymph nodes were regularly evaluated. At the study endpoint, footpad tumors together with the corresponding draining lymph nodes were harvested, processed by paraformaldehyde fixation and paraffin embedding, and sectioned for subsequent analysis. Histopathological examination using hematoxylin and eosin staining, in combination with immunohistochemical assessment, was performed to determine primary tumor characteristics and lymph node metastatic involvement.

### 4.13 Cell Proliferation Assays

Cell proliferative capacity was examined by combining the Cell Counting Kit‐8 (CCK‐8; APExBIO, USA) assay with clonogenic growth analysis, in accordance with established protocols [51, 52]. For the CCK‐8 assay, cells were dispensed into 96‐well plates at a starting density of 2 × 10^3^ cells per well and maintained under routine culture conditions. At specified time intervals, 10 µL of CCK‐8 reaction mixture was applied to each well, followed by incubation at 37°C for 2 h. The resulting colorimetric signal, reflecting cellular metabolic activity, was determined by spectrophotometric measurement at 450 nm.

For colony formation analysis, 1000–2000 cells were placed into 6‐well plates and allowed to grow for 1–2 weeks, with the culture medium renewed every 2–3 days. At the conclusion of the assay, colonies were chemically fixed using 4% paraformaldehyde for 20 min, rendered visible with 0.1% crystal violet staining, and quantified by microscopic evaluation.

### 4.14 Flow Cytometry Analysis

Apoptotic responses and cell cycle profiles were evaluated by flow cytometric approaches. For apoptosis detection, cells were labeled using an Annexin V‐FITC/propidium iodide (PI) staining system (Annexin V‐FITC/PI Apoptosis Detection Kit; Vazyme, China) following the manufacturer's guidelines [53, 54]. Briefly, cells were collected, rinsed twice with ice‐cold phosphate‐buffered saline, resuspended in binding buffer, and exposed to the staining reagents for 15 min at room temperature under light‐protected conditions prior to immediate measurement.

For cell cycle profiling, cells were processed with a Cell Cycle Detection Kit (Vazyme, China). Cells were permeabilized by fixation in 70% ethanol at 4°C overnight, followed by washing with PBS and incubation with a PI/RNase staining mixture for 30 min at room temperature in the dark. Flow cytometric acquisition was conducted on a Beckman Coulter cytometer (China), and the resulting data were processed and quantified using FlowJo software (BD, USA).

### 4.15 Copper Content Analysis

Intracellular and tissue copper (Cu^2^^+^) levels were quantified using commercially available colorimetric assay kits. For cellular measurements, normal urothelial cells and BCa cell lines were analyzed using the Cell Copper (Cu^2^^+^) Colorimetric Assay Kit (Elabscience, China). For tissue measurements, paired normal bladder and BCa specimens were assessed with the Copper (Cu^2^^+^) Colorimetric Assay Kit (Elabscience, China). All experimental procedures followed the relevant kit specifications, and copper levels were quantified by reference to the appropriate standard calibration curves.

### 4.16 Cell Viability and IC_50_ Analysis

The response of bladder cancer cells to Elesclomol combined with Cu(II) (ES‐Cu) was assessed by the Cell Counting Kit‐8 (CCK‐8) assay. UM‐UC‐3 and T24 cells, as well as their DIXDC1 knockdown or overexpression derivatives, were plated in 96‐well plates at a density of 2 × 10^3^ cells per well. Following overnight adherence, cells were treated with increasing concentrations of Elesclomol (0–100 nm) in the presence of 4 µm CuCl_2_ for 24 h. Absorbance at 450 nm was recorded using a microplate reader, and dose‐response curves were constructed. Half‐maximal inhibitory concentration (IC_50_) values were determined by nonlinear regression analysis using GraphPad Prism based on a four‐parameter logistic regression model (4PL; log[inhibitor] vs. response, variable slope), with 95% confidence intervals reported. All experiments were performed in at least three independent repeats. Because IC_50_ values were derived from nonlinear curve fitting, direct statistical comparisons of IC_50_ values between groups were not performed; differences were described based on the fitted dose‐response curves and their 95% confidence intervals.

### 4.17 Ghost Dye Flow Cytometry for Cell Death Analysis

Cell death was assessed using Ghost Dye staining followed by flow cytometry. T24 and UM‐UC‐3 cells were transfected with si‐NC or si‐DIXDC1 as indicated and then treated with DMSO, ES‐Cu, TTM, or ES‐Cu combined with TTM. ES‐Cu treatment consisted of elesclomol (30 nm) and CuCl_2_ (4 µm). For copper‐dependence rescue experiments, cells were pretreated with TTM (20 µm) for 1 h, followed by ES‐Cu treatment for 12 h in the continued presence of TTM. For cell death inhibitor and redox intervention assays, DIXDC1‐knockdown cells were pretreated with TTM (20 µm), Z‐VAD‐FMK (20 µm), Nec‐1 (20 µm), Belnacasan (VX‐765, 10 µm), Lip‐1 (0.5 µm), or DTT (0.5 mm) for 1 h, followed by ES‐Cu treatment for 12 h in the continued presence of the corresponding inhibitors or redox reagent. After treatment, both floating and adherent cells were collected, washed with PBS, and stained with Ghost Dye (1:1000; Cytek Biosciences, China) according to the manufacturer's instructions in the dark. Cells were then washed and analyzed by flow cytometry. Cell death was quantified as the percentage of Ghost Dye‐positive cells.

### 4.18 Subcutaneous Xenograft Model

Male BALB/c nude mice (4–5 weeks old, SPF grade) were randomly assigned to four experimental groups (Vector, DIXDC1, sh‐NC, and sh‐DIXDC1; *n* = 12 per group). To establish subcutaneous xenografts, 1 × 10^7^ cells in 100 µL PBS were injected into the dorsal region of the right forelimb. After tumor formation, animals were further divided into PBS control and drug‐treatment cohorts, as previously described [55]. Mice allocated to the treatment arm were administered Cu(II)‐elesclomol (Cu‐ES; Selleck, E4801, USA) by intraperitoneal delivery at 6 mg/kg on every 3 days schedule, with dosing adjustments implemented as needed to maintain tolerability [56]. Tumor growth kinetics were followed at three‐day intervals by caliper assessment, and tumor volume was estimated using the equation V = 0.5 × length × width^2^. At study termination, animals were euthanized, and tumor tissues were excised, documented by weight and imaging, and prepared for subsequent histological and immunohistochemical examinations.

### 4.19 Orthotopic Bladder Cancer Xenograft Model

Orthotopic bladder cancer xenograft models were established using luciferase‐labeled UM‐UC‐3 cells with stable DIXDC1 overexpression or knockdown, together with their respective control cells. Briefly, 4–5‐week‐old female BALB/c nude mice were anesthetized, and the bladder was exposed through a lower abdominal incision. A total of 2 × 10^6^ cells suspended in 30 µL serum‐free medium were injected into the bladder wall using an insulin syringe. Tumor growth was monitored by in vivo bioluminescence imaging every 2 days. At day 14 post‐inoculation, mice were sacrificed, and primary bladder tumors together with pelvic lymph nodes were harvested for subsequent analysis. Bladder tumor volume, bladder tumor weight, and pelvic lymph node volume were measured at the experimental endpoint.

### 4.20 TUNEL Assay

Apoptotic cells in tumor tissues were evaluated by TUNEL staining on paraffin‐embedded sections using a commercially available TUNEL Apoptosis Detection Kit (Servicebio, G1501, China), with minor modifications to the manufacturer's protocol. In brief, tissue sections were deparaffinized, rehydrated, permeabilized with proteinase K, and incubated with the TdT reaction mixture at 37°C. After nuclear counterstaining with DAPI or propidium iodide (PI), the sections were coverslipped with an anti‐fade mounting medium and visualized under a fluorescence microscope.

### 4.21 Immunofluorescence (IF) Staining

Protein subcellular localization in bladder cancer cells was examined by immunofluorescence analysis. Cells cultured on glass‐bottom confocal dishes were fixed, permeabilized, and blocked to minimize nonspecific binding. Primary antibody incubation was performed at 4°C overnight, followed by incubation with fluorophore‐labeled secondary antibodies. Nuclei were counterstained with DAPI (Servicebio, China). Fluorescence imaging was performed with a Leica SP8 confocal microscope (Leica Microsystems, Germany) under identical acquisition parameters and analyzed using ImageJ software. Colocalization analysis was performed using two approaches. First, Pearson's correlation coefficient (R value) was calculated using the Coloc 2 plugin in ImageJ to quantitatively assess the correlation between DIXDC1 and DLAT fluorescence signals. Second, fluorescence intensity‐distance profiles were extracted along selected regions using the Plot Profile function to evaluate the spatial colocalization pattern of the two proteins.

### 4.22 Statistics

Statistical processing and graphical output were completed with GraphPad Prism 11.0.2 Continuous variables are summarized as mean ± standard deviation (SD). Groupwise comparisons were carried out by selecting unpaired or paired Student's *t* tests, the Mann‐Whitney U test, one‐way ANOVA followed by Dunnett's multiple‐comparisons test, or two‐way ANOVA, depending on data structure and comparison design. For one‐way ANOVA analyses, Tukey's, Šídák's, or Dunnett's multiple‐comparisons test was used as appropriate. Associations between gene expression variables were examined through Pearson correlation analysis. Time‐to‐event outcomes were visualized by Kaplan‐Meier survival plots, with intergroup differences evaluated using the log‐rank (Mantel‐Cox) test. Univariate Cox regression analysis was first performed to evaluate the association between clinicopathological variables and survival outcomes, and variables with *p* < 0.05 in univariate analysis were further included in the multivariate Cox regression model. Hazard ratios (HRs) and 95% confidence intervals (CIs) were reported. Additional analyses were implemented in R software (version 4.4.1). A *p‐*value < 0.05 was considered indicative of statistical significance.

## Author Contributions

H.F.W., X.C., and L.C. conceived and supervised the overall study and provided strategic guidance. L.C., H.Q.L., X.L.L., and H.N.D. performed most of the experiments, analyzed the data, and drafted the manuscript. X.K. provided clinical specimens and performed pathological evaluations. S.F., H.J.S., M.S.L., X.N.Y., and X.Y.Z. assisted in clinical sample processing and data curation. C.W.Y., Y.H.H., Z.H.C., Y.W.Z., K.W.J., and S.T.C. provided experimental support and technical optimization. All authors discussed the results and approved the final manuscript.

## Ethics Statement

All bladder cancer tissues were obtained with informed consent from patients and approved by the Ethics Committee (Approval No. PJ‐Dept‐2025‐146). All animal experiments were approved by the Institutional Animal Care and Use Committee of Sun Yat‐sen University (Approval No. AP20240310).

## Conflicts of Interest

The authors declare no conflicts of interest.

## Supporting information




**Supporting File**: advs77435‐sup‐0001‐SuppMat.docx.

## Data Availability

All data supporting the conclusions of this study are included in the main text and supplementary tables. The full dataset is available in the supplementary materials.
