# Supplementary material for: DIXDC1 Promotes Lymphatic Metastasis and Resistance to Cuproptosis in Bladder Cancer Through Mediating DLAT
Source: Adv Sci (Weinh). 2026 Aug 27;13(63):e77435. doi: 10.1002/advs.77435 (PMC13521190; doi:10.1002/advs.77435)
Supplement: Supplementary file 1 [file ADVS-13-e77435-s001.docx]

**DIXDC1 promotes lymphatic metastasis and resistance to cuproptosis in bladder cancer through mediating DLAT**

Hongqiong Li^1#^	|	Liang Cheng^2, 3, 4#*^	|	Xiaole Lu^2, 3, #^	| Haonan Dong^1#^, | Shi Fu^1^	| Hongjin Shi^1^	| Mingsheng Liu^1, 5^	|	Chenwei Yang^2, 3^	|	Yuhang Huang^2, 3^	|	Zehua Chen^2, 3^	|	Yawei Zhang^1^	| Kaiwen Jie^2, 3^	| Xiaoyue Zhang^1^	|	Xiang Kui^6^	|	Xinni Ye^1^	|	Siting Chen^2, 3^	|	Xu Chen^2, 3, 4*^	|	Haifeng Wang^1*^

1 Department of Urology, The Second Affiliated Hospital of Kunming Medical University, Kunming 650101, Yunnan, P. R. China	|	2 Department of Urology, Sun Yat-sen Memorial Hospital, Sun Yat-sen University, Guangzhou 510120, Guangdong, P. R. China	|	3 Guangdong Provincial Key Laboratory of Malignant Tumor Epigenetics and Gene Regulation, Sun Yat-sen Memorial Hospital, State Key Laboratory of Oncology in South China, Guangzhou 510120, Guangdong, P. R. China	|	4 Guangdong Provincial Clinical Research Center for Urological Diseases, Guangzhou, Guangdong 510120, P. R. China	|	5 Department of Urology, Qujing Affiliated Hospital of Kunming Medical University, Qujing 655000, Yunnan, P. R. China	|	6 Department of Pathology, The Second Affiliated Hospital of Kunming Medical University, Kunming 650101, Yunnan, P. R. China

\# These authors contributed equally to this study.

*Correspondence: Haifeng Wang ([wanghaifeng@kmmu.edu.cn](mailto:wanghaifeng@kmmu.edu.cn))	|	Xu Chen ([chenx457@mail.sysu.edu.cn](mailto:chenx457@mail.sysu.edu.cn))	|	Liang Cheng (chengliang9@mail.sysu.edu.cn)

**Supplementary figure legends**

**Supplementary Figure 1	|	DIXDC1 is overexpressed and associated with poor prognosis in bladder cancer.** (A and B) Quantitative H-score analysis of DIXDC1 protein expression in low-grade and high-grade bladder cancer tissues from Cohort 1 (A) and Cohort 2 (B). (C) Analysis of *DIXDC1* mRNA expression in low-grade and high-grade bladder cancer samples from The Cancer Genome Atlas (TCGA) database. (D) Comparison of *DIXDC1* mRNA expression between early-stage (T1-T2) and advanced-stage (T3-T4) bladder cancer samples based on the TCGA database. (E) Analysis of DIXDC1 expression differences among bladder cancer samples with distinct T stages (T1-T2 vs T3-T4) using the GSE87304 dataset. (F and G) Comparison of DIXDC1 protein expression in tumor tissues from bladder cancer patients without distant metastasis (M0) and with distant metastasis (M1) in Cohort 1 (F) and Cohort 2 (G). (H and I) Kaplan-Meier survival analysis of overall survival (H) and disease-free survival (I) in bladder cancer patients from the TCGA cohort, stratified by high and low DIXDC1 expression. Data are presented as means ± standard deviations (SD). Statistical analyses were performed using the unpaired Student’s t-test for panels A-B and F-G, the Mann-Whitney U test for panels C-E, and the log-rank (Mantel-Cox) test for panels H-I.

**Supplementary Figure 2	|	DIXDC1 promotes the metastasis-related behaviours of bladder cancer cells *in vitro* and enhances lymph node metastasis *in vivo*.** (A and B) Following the overexpression (A) and knockdown (B) of DIXDC1, qRT-PCR was employed to quantify the mRNA levels of DIXDC1 in T24 and UM-UC-3 cells. (C) Following the overexpression and knockdown of DIXDC1, representative images and quantitative bar graphs of the wound healing assay were obtained in T24 and UM-UC-3 cells. Scale bar: white, 200 μm. (D) Representative images of H&E staining of popliteal lymph nodes, along with the lymph node metastasis rates in different groups, are presented. (E and F) Representative images of GFP immunohistochemical staining (E) and quantitative analysis of GFP expression based on GFP H-score (F) in popliteal lymph nodes from each experimental group. Scale bar: black, 400 μm; red, 100 μm; blue, 100 μm. Data are expressed as means ± standard deviations (SD). Statistical analyses were performed using unpaired Student’s t-tests for panel A, the upper graphs in panel C, and panel F, and one-way ANOVA followed by Dunnett’s multiple-comparisons test for panel B and the lower graphs in panel C.

**Supplementary Figure 3	|	DIXDC1 modulates the migration of bladder cancer cells via DLAT.** (A and B) Colocalization analysis between DIXDC1 and DLAT in T24 cells, with Pearson correlation scatter plot for quantifying fluorescence signal overlap between DIXDC1 and DLAT (A) and line scan analysis for assessing their spatial fluorescence intensity distribution along the selected cellular region (B), as assessed by confocal immunofluorescence microscopy. (C and D) Detection of *DLAT* mRNA levels in T24 and UM-UC-3 cells following DIXDC1 overexpression (C) and silencing (D) by qRT-PCR. (E-H) Transwell migration and invasion assays in T24 and UM-UC-3 cells following DLAT knockdown in the context of DIXDC1 overexpression, with quantification of migrated cells (E), quantification of invaded cells (F), representative migration images (G), and representative invasion images (H). Scale bar: 200 μm (red). (I-K) Wound healing assays in DIXDC1-overexpressing T24 and UM-UC-3 cells following DLAT knockdown, with quantification of wound healing percentage (I), representative images of T24 cells (J), and representative images of UM-UC-3 cells (K). Scale bar: 200 μm (white). Data are presented as means ± standard deviations (SD). Statistical analyses were performed using Pearson correlation analysis for panel A, unpaired Student’s t-tests for panel C, and one-way ANOVA followed by Dunnett’s multiple-comparisons test for panels D-F and I.

**Supplementary Figure 4	|	DLAT is overexpressed and associated with poor prognosis in bladder cancer.** (A) Representative immunohistochemical (IHC) staining images showing DLAT expression in adjacent normal tissues (NAT), lymph node-negative bladder cancer tissues (LN⁻ CA), and lymph node-positive bladder cancer tissues (LN⁺ CA), with high-magnification images of the red-framed regions shown below. Scale bar: 100 μm (black), 20 μm (red). (B and C) Quantitative H-score analysis of DLAT protein expression in NAT, LN⁻ CA, and LN⁺ CA tissues from Cohort 1 (B) and Cohort 2 (C). (D and E) Quantitative H-score analysis of DLAT protein expression in non-muscle-invasive bladder cancer (NMIBC) and muscle-invasive bladder cancer (MIBC) tissues from Cohort 1 (D) and Cohort 2 (E). (F) Quantitative H-score analysis of DLAT protein expression in bladder cancer tissues at M0 and M1 stages from Cohort 1. (G) Analysis of *DLAT* mRNA expression in high- and low-grade bladder cancer samples based on The Cancer Genome Atlas (TCGA) database. (H and I) Kaplan-Meier survival analysis of overall survival (H) and disease-free survival (I) in bladder cancer patients from Cohort 1, stratified by high and low DLAT expression. (J and K) Kaplan-Meier survival analysis of overall survival (J) and disease-free survival (K) in bladder cancer patients from Cohort 2, stratified by high and low DLAT expression. Data are presented as means ± standard deviations (SD). Statistical analysis was performed using the ordinary one-way ANOVA followed by Tukey’s multiple-comparisons test for panels B to C, unpaired Student’s t-test for panels D to F; the Mann-Whitney U test for panel G; and the log-rank (Mantel-Cox) test for panels H to K.

**Supplementary Figure 5	|	Inhibition of DLAT impairs the migration, invasion, and proliferation of bladder cancer cells.** (A) Detection of *DLAT* mRNA expression in T24 and UM-UC-3 cells following DLAT knockdown by qRT-PCR. (B) Western blot analysis of DLAT protein expression in T24 and UM-UC-3 cells with DLAT knockdown. (C-G) Representative images and corresponding quantitative bar graphs (F and G) of wound healing assays (C), migration assays (D), and invasion assays (E) in T24 and UM-UC-3 cells following DLAT knockdown. Scale bars: 200 μm (white); 200 μm (red). (H and I) Cell proliferation curves of T24 (H) and UM-UC-3 (I) cells measured by CCK-8 assay after DLAT knockdown. (J and K) Representative images (J) and corresponding quantitative bar graphs (K) of colony formation assays in T24 and UM-UC-3 cells with DLAT knockdown. Data are presented as means ± standard deviations (SD). Statistical analyses were performed using one-way ANOVA followed by Dunnett’s multiple-comparisons test for panels A, C, F, G, and K, and two-way ANOVA followed by Dunnett’s multiple-comparisons test for panels H and I.

**Supplementary Figure 6 |	NEK7 and CCNE2 mediate DIXDC1-regulated migration and invasion in bladder cancer cells.** (A and B) Transwell migration and invasion assays in T24 and UM-UC-3 cells following DIXDC1 knockdown, with or without CCNE2 or NEK7 overexpression, with representative migration images (A) and representative invasion images (B). Scale bars: 200 μm (red).

**Supplementary Figure 7 |	DIXDC1 promotes proliferation and confers resistance to cuproptosis in bladder cancer cells *in vitro*.** (A and B) Representative flow cytometry plots (A) and corresponding quantitative bar graphs (B) for cell cycle distribution analysis in DIXDC1-knockdown T24 cells. (C and D) Representative flow cytometry plots (C) and corresponding quantitative bar graphs (D) for cell cycle distribution analysis in DIXDC1-knockdown UM-UC-3 cells. (E) Representative flow cytometry plots for cell cycle distribution analysis in DIXDC1-overexpressing T24 and UM-UC-3 cells. (F and G) Corresponding quantitative bar graphs for cell cycle distribution analysis in DIXDC1-overexpressing T24 (F) and UM-UC-3 (G) cells. (H and I) Cell proliferation curves of ES-Cu-treated T24 (H) and UM-UC-3 (I) cells, as measured by CCK-8 assay, with half-maximal inhibitory concentration (IC₅₀) values calculated accordingly. All data are presented as means ± standard deviations (SD). Statistical analysis was performed using two-way analysis of variance (ANOVA) with appropriate multiple-comparison correction for panels B, D, F, and G.

**Supplementary Figure 8	|	Representative flow cytometry gating strategies for cell-cycle and Ghost Dye cell death analyses.** (A-D) Representative gating strategy for cell-cycle analysis, including initial cell population selection based on FSC-A/SSC-A to exclude debris (A), single-cell gating based on FSC-H/FSC-A to exclude doublets (B), PE-A subset selection (C), and representative PE-A DNA-content histogram analysis (D). (E-G) Representative gating strategy for Ghost Dye cell death analysis, including intact cell selection based on FSC-H/SSC-H (E), single-cell gating based on FSC-H/FSC-A (F), and quantification of Ghost Dye-positive dead cells (G).

**Supplementary Figure 9 |	DIXDC1 modulates the growth of bladder cancer cells and resistance to cuproptosis *in vivo*.** (A-B) Representative H&E and Ki67 staining images of xenograft tumors derived from UM-UC-3 cells with stable DIXDC1 overexpression (A) or knockdown (B) treated with PBS or ES-Cu. Scale bar: 100 μm (black).

**Supplementary Figure 10	|	DIXDC1 regulates tumor death in bladder cancer *in vivo*.** (A) Representative TUNEL staining (green), DAPI nuclear staining (blue), and merged images of xenograft tumor tissues from DIXDC1-overexpressing UM-UC-3 cells and control cells after PBS or ES-Cu treatment. (B) Representative TUNEL staining (green), DAPI nuclear staining (blue), and merged images of xenograft tumor tissues from DIXDC1-knockdown UM-UC-3 cells and control cells with PBS or ES-Cu treatment. Scale bar: 100 μm (white).

**Supplementary Figure 11	|	DIXDC1 modulates the growth of bladder cancer cells and resistance to cuproptosis *in vivo***. (A) Representative immunohistochemical staining images of DIXDC1, DLAT, CCNE2, and NEK7 in xenograft tumor tissues with distinct DIXDC1 expression statuses and their corresponding control groups. Scale bar: 100 μm (black).

**Supplementary Figure 12	|	Histological assessment of liver and kidney tissues after ES-Cu treatment.** (A-B) Representative H&E-stained images of xenograft tumors derived from UM-UC-3 cells with stable DIXDC1 overexpression (A) or knockdown (B) treated with PBS or ES-Cu. Scale bar: 100 μm (black).

**Supplementary Table S1	|	Clinicopathological characteristics of patients in two independent cohorts**

| **Variables** | **Cohort 1 (n = 95)** | **Cohoret 2 (n = 54)** | ***p*-value** |
| --- | --- | --- | --- |
| DIXDC1 H-score  (median [IQR]) | 152.66 (123.97-188.07) | 152.60 (126.24-190.01) | 0.903 |
| DLAT H-score  (median [IQR]) | 164.13 (138.17-207.32) | 147.66 (113.22-187.01) | 0.013 |
| Gender |  |  |  |
| Female (%) | 15.00 (15.80) | 11.00 (20.40) | 0.629 |
| Male (%) | 80.00 (84.20) | 43.00 (79.60) |  |
| Age (median [IQR], y) | 62.00 (56.00-71.00) | 67.00 (59.00-73.00) | 0.087 |
| Histological type (%) |  |  |  |
| NMIBC | 26.00 (27.40) | 14.00 (25.90) | 1.000 |
| MIBC | 69.00 (72.60) | 40.00 (74.10) |  |
| pN stage (%) |  |  |  |
| LN- | 46.00 (48.40) | 36.00 (66.70) | 0.048 |
| LN+ | 49.00 (51.60) | 18.00 (33.30) |  |
| pM stage (%) |  |  |  |
| M- | 76.00 (80.00) | 41.00 (75.90) | 0.048 |
| M+ | 19.00 (20.0) | 13.00 (24.10) |  |
| Grade (%) |  |  |  |
| Low grade | 19.00 (20.00) | 23.00 (42.60) | 0.006 |
| High grade | 76.00 (80.00) | 31.00 (57.40) |  |

Continuous variables are presented as median (IQR) and categorical variables as number (percentage). Group comparisons were performed using the Mann-Whitney U test, chi-square test, or Fisher’s exact test, as appropriate. Cohort 1 was from the Second Affiliated Hospital of Kunming Medical University and Cohort 2 from Sun Yat-sen Memorial Hospital. Abbreviations: IQR, interquartile range; NMIBC, non-muscle-invasive bladder cancer; MIBC, muscle-invasive bladder cancer; pN, pathological lymph node stage; pM, pathological metastasis stage.

**Supplementary Table S2	|	Univariate and multivariate Cox regression analyses of prognostic factors for overall survival in the combined cohort (cohort 1 and cohort 2)**

| **Variables** | **Univariate analysis** | | | **Multivariate analysis** | | |
| --- | --- | --- | --- | --- | --- | --- |
|  | **HR** | **95% CI** | ***P*** | **HR** | **95% CI** | ***P*** |
| DIXDC1 H-score  (≤214.86 / > 214.86) | 6.54 | 3.46-12.34 | **<0.0001** | 3.27 | 1.51-7.10 | **0.0030** |
| DLAT H-score  (212.42 / > 212.42) | 3.61 | 1.98-6.60 | **<0.0001** | 1.72 | 0.86-3.43 | 0.1260 |
| Gender (Male / Female) | 1.63 | 0.73-3.64 | 0.2351 |  |  |  |
| Age (≤73 / >73) | 2.85 | 1.48-5.51 | **0.0018** | 1.65 | 0.78-3.49 | 0.1930 |
| Histological type  (MIBC / NMIBC) | 3.77 | 1.60-8.88 | **0.0024** | 1.99 | 0.76-5.23 | 0.1630 |
| pN stage (LN- / LN-) | 2.07 | 1.16-3.68 | **0.0136** | 1.23 | 0.63-2.40 | 0.5340 |
| pM stage (M+ / M-) | 3.62 | 1.97-6.65 | **<0.0001** | 1.92 | 0.97-3.82 | 0.0620 |
| Grade  (Low grade / High grade) | 2.85 | 1.33-6.10 | **0.0070** | 1.29 | 0.52-3.19 | 0.5880 |

Variables with *p*< 0.05 in univariate analysis were included in the multivariate Cox model. Hazard ratios (HRs) and 95% confidence intervals (CIs) were estimated using Cox proportional hazards regression. Significant *p*-values are shown in bold. HR > 1 indicates increased risk of death, whereas HR < 1 indicates reduced risk. Continuous variables were dichotomized using optimal cutoff values determined by maximally selected rank statistics. Analyses were conducted on the pooled dataset from cohort 1 and cohort 2.

**Supplementary Table S3	|	Univariate and multivariate Cox regression analyses of prognostic factors for disease-free survival in the combined cohort (cohort 1 and cohort 2)**

| **Variables** | **Univariate analysis** | | | **Multivariate analysis** | | |
| --- | --- | --- | --- | --- | --- | --- |
|  | **HR** | **95% CI** | ***P*** | **HR** | **95% CI** | ***P*** |
| DIXDC1 H-score  (≤214.86 / > 214.86) | 3.73 | 1.99-6.99 | **<0.0001** | 2.11 | 0.96-4.64 | 0.0620 |
| DLAT H-score  (≤212.42 / > 212.42) | 2.38 | 1.32-4.28 | **0.0039** | 1.29 | 0.64-2.57 | 0.4780 |
| Gender (Male / Female) | 1.48 | 0.74-2.95 | 0.2647 |  |  |  |
| Age (≤73 / >73) | 2.56 | 1.37-4.79 | **0.0033** | 1.92 | 0.96-3.84 | 0.0640 |
| Histological type  (MIBC / NMIBC) | 1.76 | 0.96-3.22 | 0.0654 | 1.19 | 0.59-2.39 | 0.6360 |
| pN stage (LN- / LN-) | 2.41 | 1.42-4.12 | **0.0012** | 2.01 | 1.07-3.78 | **0.0310** |
| pM stage (M+ / M-) | 2.42 | 1.32-4.45 | **0.0044** | 1.39 | 0.70-2.76 | 0.3530 |
| Grade  (Low grade / High grade) | 1.83 | 1.00-3.35 | **0.0492** | 0.97 | 0.46-2.08 | 0.9460 |

Variables with *p* < 0.05 in univariate analysis were included in the multivariate Cox model. Hazard ratios (HRs) and 95% confidence intervals (CIs) were estimated using Cox proportional hazards regression. Significant *p*-values are shown in bold. HR > 1 indicates increased risk of recurrence, whereas HR < 1 indicates reduced risk. Continuous variables were dichotomized using optimal cutoff values determined by maximally selected rank statistics. Analyses were conducted on the pooled dataset from cohort 1 and cohort 2.

**Supplementary Table S4|	Antibodies used in this study.**

| **Application** | **Protein or histone modification** | **Manufacturer** | **Cat number** | **Dilution for usage** |
| --- | --- | --- | --- | --- |
| Western blotting | DIXDC1 | Abcam | Ab226210 | 1:1000 |
| Western blotting | DLAT | Proteintech | 13426-1-AP | 1:1000 |
| Western blotting | CCNE2 | Proteintech | 11935-1-AP | 1:1000 |
| Western blotting | NEK7 | Proteintech | 31422-1-AP | 1:1000 |
| Western blotting | FLAG | Ray Antibody | RM1002 | 1:1000 |
| Western blotting | Ub | CST | -YM3636 | 1:1000 |
| Western blotting | GAPDH | Proteintech | 60004-1-Ig | 1:10000 |
| Western blotting | IgG | CST | #38066 | 1:1000 |
| Western blotting | HA | Proteintech | 51064-2-AP | 1:5000 |
| Western blotting | Mcherry | Proteintech | 26765-1-AP | 1:1000 |
| Immunohistochemistry | GFP | CST | #2555 | 1:1500 |
| Immunohistochemistry | Ki67 | ZSGB-BIO | ZM-0167-3 | 1:200 |
| Immunohistochemistry | DIXDC1 | Proteintech | 13816-1-AP | 1:500 |
| Immunohistochemistry | DLAT | Proteintech | 13426-1-AP | 1:500 |
| Immunohistochemistry | CCNE2 | Proteintech | 11935-1-AP | 1:800 |
| Immunohistochemistry | NEK7 | Proteintech | 31422-1-AP | 1:500 |
| Immunoprecipitation | IgG | CST | #3900 | 1:500 |
| Immunoprecipitation | DIXDC1 | Abcam | Ab226210 | 1:50 |
| Immunoprecipitation | DLAT | CST | 12362S | 1:50 |
| Immunofluorescence | DIXDC1 | DIXDC1 | Abcam | 1:50 |
| Immunofluorescence | DLAT | DLAT | CST | 1:50 |
| Immunofluorescence | DAPI | Servicebio | G1012 | 1:10 |

**Supplementary Table S5	|	Sequences of siRNA oligos and shRNAs**

| **Name** | **Sequence 5’-3’** |
| --- | --- |
| **siRNA** |  |
| si-NC | UUCUCCGAACGUGUCACGUTT |
| si-DIXDC1#1 | GCAGCAGUACGAAGGGCAATT |
| si-DIXDC1#2 | GCAAAGAGCGAGUCCAUUATT |
| si-DLAT#1 | GCCUGGAGGAGUGUUAUAUTT |
| si-DLAT#2 | GGCUCCUGGUAGCUCAUAUTT |
| **shRNA** |  |
| sh-NC | CAACAAGATGAAGAGCACCAA |
| sh-DIXDC1#1 | GCAGCAGTACGAAGGGCAA |
| sh-DIXDC1#2 | GCAAAGAGCGAGTCCATTA |
| sh-DLAT | GCCTGGAGGAGTGTTATAT |

**Supplementary Table S6	|	Primers used in this study**

| **Primer Name** | **Sequence 5’-3’** |
| --- | --- |
| **Used in qPCR** |  |
| *DIXDC1* Forward | AGTCAGCAACAGAGGGACCAG |
| *DIXDC1* Reverse | CGCTCCATGAGGCTAGAAATG |
| *DLAT* Forward | GTTGATGTCAGTGTTGCGGT |
| *DLAT* Reverse | CCCTCTCTTGCTTTGGTTGC |
| *CCNE2* Forward | TCAAGACGAAGTAGCCGTTTAC |
| *CCNE2* Reverse | TGACATCCTGGGTAGTTTTCCTC |
| *NEK7* Forward | GCAGATGCTGGCGACCTATC |
| *NEK7* Reverse | CCCAGTGGCTGTAATGAACAC |
| *18S* Forward | ACACGGACAGGATTGACAGA |
| *18S* Reverse | GGACATCTAAGGGCATCACA |
| *GAPDH* Forward | GTCTCCTCTGACTTCAACAGCG |
| *GAPDH* Reverse | ACCACCCTGTTGCTGTAGCCAA |

**Supplementary Table S7|	High-confidence interactors from Co-IP-MS**

| Coverage | Total Peptides | PSMs | Unique Peptides | AAs | MW  [kDa] | calc.pI | Score Seq | Gene Sym |
| --- | --- | --- | --- | --- | --- | --- | --- | --- |
| 25.0% | 48 | 196 | 36 | 645 | 69.14 | 8.11 | 1754.28 | DLAT |
| 20.3% | 31 | 125 | 23 | 522 | 53.33 | 9.52 | 1126.71 | FUS |
| 17.1% | 26 | 98 | 19 | 597 | 65.27 | 8.94 | 942.35 | HNRNPAB |
| 35.2% | 22 | 87 | 17 | 335 | 36.06 | 8.58 | 815.62 | GAPDH |
| 19.6% | 21 | 79 | 15 | 510 | 55.82 | 9.17 | 752.18 | HNRNPH3 |
| 14.8% | 18 | 66 | 13 | 575 | 61.39 | 8.73 | 688.44 | DDX3X |
| 16.5% | 15 | 58 | 11 | 497 | 52.65 | 9.35 | 591.27 | FAM120A |
| 28.7% | 13 | 49 | 9 | 261 | 30.12 | 10.21 | 463.82 | RPS4X |
